# Progress of AI-Driven Drug–Target Interaction Prediction and Lead Optimization

**DOI:** 10.3390/ijms262010037

**Published:** 2025-10-15

**Authors:** Qiqi Wang, Boyan Sun, Yunpeng Yi, Tony Velkov, Jianzhong Shen, Chongshan Dai, Haiyang Jiang

**Affiliations:** 1 State Key Laboratory of Veterinary Public Health and Safety, College of Veterinary Medicine, China Agricultural University, Beijing 100193, China; 2 Technology Innovation Center for Food Safety Surveillance and Detection (Hainan), Sanya Institute of China Agricultural University, Sanya 572025, China; 3 Shandong Provincial Animal and Poultry Green Health Products Creation Engineering Laboratory, Institute of Poultry Science, Shandong Academy of Agricultural Science, 202 Gongyebeilu Jinan, Jinan 250023, China; 4 Department of Pharmacology, Biodiscovery Institute, Monash University, Clayton, VIC 3800, Australia

**Keywords:** artificial Intelligence, drug discovery, machine learning, virtual screening

## Abstract

In modern pharmaceutical research and development (R&D), drug discovery remains a challenging process. Artificial intelligence (AI) has been extensively incorporated into various phases of drug discovery and development. AI enable effectively extract molecular structural features, perform in-depth analysis of drug–target interactions, and systematically model the relationships among drugs, targets, and diseases. These approaches improve prediction accuracy, accelerate discovery timelines, reduce costs from trial and error methods, and enhance success probabilities. This review summarizes recent advances in AI applications for drug design, including target identification, synthetic accessibility prediction, lead optimization, and ADMET property evaluation. Furthermore, it introduces various deep learning tools to guide researchers in selecting and implementing the most appropriate AI-driven strategies throughout the drug discovery process. We hope it can establish a conceptual framework intended to advance AI-driven methodologies in pharmaceutical research by comprehensively organizing novel perspectives and critical insights.

## 1. Introduction

The traditional drug discovery paradigm faces formidable challenges characterized by lengthy development cycles, prohibitive costs, and high preclinical trial failure rate [1]. The process from lead compound identification to regulatory approval typically spans over 12 years with cumulative expenditures exceeding $2.5 billion [2]. Clinical trial success probabilities decline precipitously from Phase I (52%) to Phase II (28.9%), culminating in an overall success rate of merely 8.1% [3,4,5,6,7]. Global efforts are intensifying to address the persistent inefficiency challenges in drug development. The strategy involves diversifying therapeutic targets to overcome the limitations of traditional approaches. This aims to reduce the preclinical attrition rate of candidate drugs, improve R&D efficiency, and optimize the cost-effectiveness ratio to alleviate the burden of high investment.

**Figure 1:**
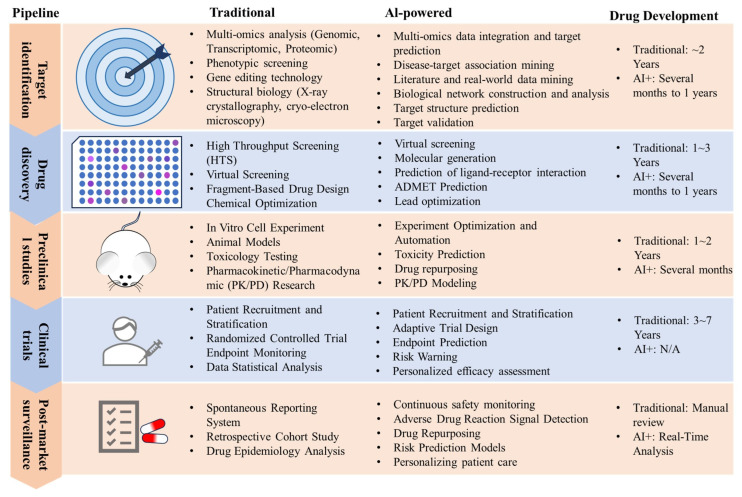
Comparative analysis of AI applications across multistage drug development pipelines.

Computational molecular modeling has catalyzed a paradigm shift in pharmaceutical research. It enables the precise simulation of receptor–ligand interactions and the optimization of lead compounds [8,9]. For example, Talukder et al. integrated molecular docking, QSAR, and molecular dynamics to identify phytochemical inhibitors targeting EGFR in non-small cell lung cancer [10]. Similarly, Kaur et al. designed blood–brain barrier (BBB) permeable β-secretase enzyme (BACE-1) inhibitors for Alzheimer’s disease using 2D-QSAR and ADMET profiling. The novel molecules exhibited good potency, BBB permeability, excellent binding affinity, and stable conformations with BACE-1 [11]. Souza et al. investigated the ability of Artificial Intelligence (AI) to balance binding affinity with drug-likeness in the design of SARS-CoV-2 protease inhibitors using machine learning (ML) [12]. Moreover, Maliyakkal et al. employed QSAR-driven virtual screening to identify *Trypanosoma cruzi* inhibitors with high predicted efficacy [13]. This paradigm shift has catalyzed the rise of AI-driven drug discovery (AIDD). ML integrates multiple omics data and structural biology insights to provide information for experimental design. Modern drug development workflows increasingly rely on these predictive systems for critical tasks including target prioritization [14,15,16], high-throughput compound screening [17,18,19,20], synthetic route planning [21,22,23], and polymorph screening [24,25,26] (Figure 1). A representative research achievement from Insilico Medicine is rentosertib, an AI-discovered drug that has completed Phase II trials for pulmonary fibrosis, showcasing the power of its AI platform [27]. Parallel advancements in antimicrobial discovery reveal that computational drug design platforms can systematically decode structural determinants of antibiotic efficacy, particularly against drug resistant pathogens [28]. The field’s transformative potential was further validated by the 2024 Nobel Prize in Chemistry, awarded for AI-powered innovations in protein engineering [29].

AIDD has experienced rapid advances, and Table 1 presents representative AI-designed small molecules currently progressing through clinical trials. A key technological strength lies in the capacity to decode intricate structure-activity relationships, facilitating de novo generation of bioactive compounds with optimized pharmacokinetic properties. The efficacy of these algorithms is intrinsically linked to the quality and volume of training data, particularly in deciphering latent patterns within complex biological datasets [30,31,32,33]. As deep learning architectures continue to evolve, the integration of AIDD into pharmaceutical pipelines is poised to significantly enhance development efficiency and substantially reduce costs.

**Table 1:** Selected examples of AI-discovered/designed small molecules in clinical stages.

Small Molecule	Company	Target	Stage	Indication
REC-1245 [34]	Recursion	RBM39	Phase 1	Biomarker-enriched solid Tumors and lymphoma
REC-3565 [34]	Recursion	MALT1	Phase 1	B-Cell Malignancies
REC-4539 [34]	Recursion	LSD1	Phase 1/2	Small-Cell Lung Cancer
REC-4881 [34]	Recursion	MEK Inhibitor	Phase 2	Familial adenomatous polyposis
REC-3964 [34]	Recursion	Selective C. diff Toxin Inhibitor	Phase 2	Clostridioides difficile Infection
REC-7735 [34]	Recursion	PI3Kα H1047R	Preclinical	HER2−HR+ Breast cancer
REV102 [34]	Recursion	ENPP1	Candidate profiling	Hypophosphatasia
ISM-6631 [35]	Insilico Medicine	Pan-TEAD	Phase 1	Mesothelioma, and Solid Tumors
ISM-3412 [35]	Insilico Medicine	MAT2A	Phase 1	MTAP−/− Cancers
INS018-055 [35]	Insilico Medicine	TNIK	Phase 2a	IPF
ISM-3091 [35]	Insilico Medicine	USP1	Phase 1	BRCA mutant cancer
ISM-8207 [35]	Insilico Medicine	QPCTL	Phase 1	Solid Tumors
ISM-5043 [35]	Insilico Medicine	KAT6	Phase 1	Breast cancer
ISM-5939 [35]	Insilico Medicine	ENPP1	IND Clearance	Solid Tumors
ISM-5411 [35]	Insilico Medicine	PHD	Phase 1	IBD/Anemia of CKD
ISM-3312 [35]	Insilico Medicine	3CLpro	Phase 1	COVID-19
RLY-4008 [36]	Relay Therapeutics	FGFR2	Phase 1/2	FGFR2-altered cholangiocarcinoma
RLY-8161 [36]	Relay Therapeutics	NRAS	Preclinical	Solid Tumors
RLY-2608 [36]	Relay Therapeutics	PI3Kα	Phase 1/2	Advanced Breast Cancer
EXS4318 [37]	Exscientia	PKC-theta	Phase 1	Inflammatory and immunologic diseases
GTAEXS617 [37]	Exscientia	CDK7	Phase 1/2	Solid Tumors
SIGX-1094 [38]	Signet Therapeutics	FAK	Phase 1/2	Solid Tumors
BG-89894 [39]	BeiGene	Mat2A	Phase 1	Advanced Solid Tumors
H002 [40]	RedCloud Bio	EGFR	Phase 1	Non-Small Cell Lung Cancer
AC0682 [41]	Accutar Biotech	ER Degrader	Phase 1	Breast Cancer
AC0682 [41]	Accutar Biotech	ER Degrader	Phase 1	Breast Cancer
AC0176 [41]	Accutar Biotech	AR Degrader	Phase 1	Prostate Cancer
AC0676 [41]	Accutar Biotech	BTK Degrader	Phase 1	Hematology Oncology Indications
MDR-001 [42]	MindRank	GLP-1	Phase 1/2	Obesity/Type 2 Diabetes Mellitus
DF-006 [43]	Drug Farm	ALPK1	Phase 1	Hepatitis B/Hepatocellular cancer
LAM-001 [44]	OrphAI Therapeutics	mTOR	Phase 2	PH/BOS
HLX-1502 [45]	Healx	N/A ^b^	Phase 2	Neurofibromatosis Type 1
BGE-105 [46]	BioAge	APJ agonist	Phase 2	Obesity/Type 2 diabetes
BXCL501 [47]	BioXcel Therapeutics	alpha-2 adrenergic	Phase 2/3	Neurological Disorders
EVX-01 [48]	Evaxion Biotech	N/A ^b^	Phase 2	Metastatic melanoma
EVX-02 [48]	Evaxion Biotech	N/A ^b^	Phase 1	Adjuvant melanoma

^b^, no marked information.

This review provides a systematic overview of recent advances in AI for medicinal chemistry design. While previous surveys have broadly outlined AI applications across the entire drug discovery pipeline, this work focuses specifically on two pivotal stages: drug–target interaction (DTI) prediction and lead optimization. We summarize a range of deep learning (DL) tools to assist researchers in selecting and implementing suitable AI-driven strategies within the drug discovery process. In addition to the core focus on DTI and lead optimization, discussions were included on target identification, synthetic feasibility prediction, virtual screening, and ADMET evaluation. It demonstrates that integrating these phases with AI reduces false positives, improves compound prioritization, and accelerates therapeutic design. The discussion also highlights the translational impact of current methods and identifies promising directions for future development.

## 2. AI Technology

AI develops systems capable of human-like reasoning and decision-making. Contemporary AI systems integrate ML and DL to address pharmaceutical challenges ranging from target validation to formulation optimization.

ML employs algorithmic frameworks to analyze high-dimensional datasets, identify latent patterns, and construct predictive models through iterative optimization processes [49]. ML has evolved into four principal paradigms: supervised learning, unsupervised learning, semi-supervised learning, and reinforcement learning [50]. Supervised learning employs labeled datasets for classification via algorithms like support vector machines (SVMs) and for regression via algorithms like support vector regression (SVR) and random forests (RFs) [51,52]. Unsupervised learning identifies latent data structures through clustering and dimensionality reduction techniques (such as principal component analysis and K-means clustering) to reveal underlying pharmacological patterns and streamline chemical descriptor analysis [53,54]. In contrast, t-distributed stochastic neighbor embedding (t-SNE) primarily serves as a nonlinear visualization tool, effectively mapping high-dimensional molecular features into low-dimensional spaces to facilitate the interpretation of chemical similarity and class separation [55]. Semi-supervised learning boosts drug–target interaction prediction by leveraging a small set of labeled data alongside a large pool of unlabeled data. This is achieved through model collaboration and by generating simulated data, which enhances prediction reliability [56,57]. Reinforcement learning optimizes molecular design via Markov decision processes, where agents iteratively refine policies to generate inhibitors and balance pharmacokinetic properties through reward-driven strategies [58,59]. A comparison of ML models are shown in Table 2.

Building upon these models, a broad spectrum of shallow learning models (such as SVM, decision Trees, RF, and artificial neural networks) has historically served as the computational backbone of data-driven drug discovery. These models provide strong interpretability, efficient training on moderate-sized datasets, and robust performance for QSAR modeling, activity prediction, and ADMET estimation. Their comparative strengths and limitations are summarized in Table 3, which outlines their respective mechanisms, advantages, and representative applications across medicinal chemistry tasks.

**Figure 2:**
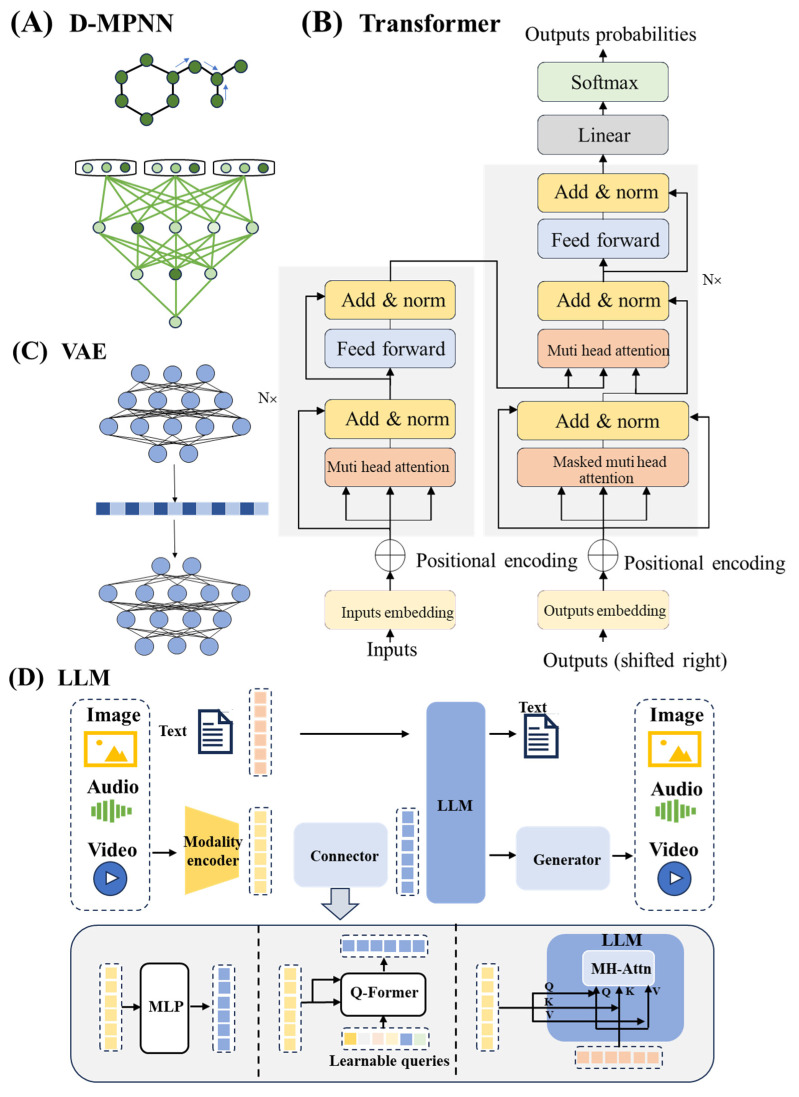
Deep learning model architectures. (**A**), Directed message-passing neural network architecture [102]. (**B**), Transformer model architecture. Key hyperparameters include: N denotes the number of encoder/decoder layers, Muti-head attention represents the attention heads, and the dimension of input embeddings represents the length of the continuous, dense vector to which each discrete input token [104]. (**C**), VAE model architecture. (**D**), LLM model architecture. LLM processes diverse inputs through modality-specific encoders that convert heterogeneous data into latent representations. Cross-modal alignment is achieved via a query-based transformer (Q-Former) employing multi-head attention (MH-Attn) mechanisms. The central LLM performs joint reasoning enhanced by MLP-based feature fusion, and multimodal generators synthesize coordinated outputs such as text or images, completing the comprehension–generation cycle.

Despite the success of shallow learning models, the representational capacity of shallow models is limited when dealing with complex, nonlinear biological data or multimodal molecular information. This limitation has driven the evolution toward DL architectures. DL, as a subset of ML, processes information through multilayer neural networks. These networks automatically learn a hierarchy of features directly from raw data via a sequence of functional layers (e.g., convolutional layers, self-attention layers). The process begins with initial layers capturing low-level, local patterns (such as atomic properties in a molecule or edges in an image). These elementary features are then progressively combined and transformed through subsequent layers to form higher-level, more abstract representations. This multi-stage, hierarchical processing is powered by nonlinear transformations introduced by activation functions at each step, enabling DL models to approximate highly complex, nonlinear relationships that are inherent in multidimensional biological and chemical data [62]. Different models have different core mechanisms. Convolutional neural networks (CNNs) are designed to analyze grid-like data, owing to their unique convolutional architecture. This capability makes them particularly valuable for biomedical image processing tasks, such as cell morphology analysis [63]. Recurrent neural networks (RNNs), with their internal recurrent connections, effectively model sequential data. For instance, they can process SMILES strings for automated molecular generation [64]. Although RNNs once represented the state-of-the-art in this domain, they have gradually been supplanted by Transformer-based architectures. Transformers excel because they more effectively capture long-range dependencies and contextual relationships in complex datasets. Nonetheless, RNNs still demonstrate advantages in modeling local sequential patterns [65]. Graph neural networks (GNNs) analyze graph structures by propagating neighborhood information, excelling at molecular property prediction (Figure 2A) [66,67]. In contrast, Transformer models use self-attention to capture long-range dependencies in sequences, showing great promise for innovative tasks like designing antiviral analogs (Figure 2B) [66,67,68,69,70].In terms of new molecule generation, Generative adversarial networks (GANs) synthesize structurally diverse candidate molecules through an adversarial training framework involving a generator and a discriminator [71,72,73,74]. Variational autoencoders (VAEs) explore molecular structures within a latent space using an encoder–decoder architecture (Figure 2C) [75,76,77,78,79]. Large language models (LLMs) have shown remarkable capabilities in handling massive multimodal data, and their potential in generative drug design is being gradually uncovered. These models are expected to assist drug discovery by integrating structured knowledge extracted from scientific literature (Figure 2D) [80,81,82,83]. It should be noted that their direct application to generative drug design remains nascent. In the short term, their more established potential lies in supporting rational drug discovery workflows through literature mining, hypothesis generation, and knowledge integration [84]. These diverse DL architectures collectively aim to address critical bottlenecks in drug research, powerfully supporting core tasks such as toxicity prediction, molecular property optimization, and de novo drug design (for a detailed comparative summary of various models, see Table 4).

In practice, many studies employ the same public datasets (such as MoleculeNet, TDC) to ensure fair model assessment and to compare results with prior work [85,86]. However, differences in research objectives, model architectures, and evaluation strategies often lead to diverse reporting practices. Increasingly, hybrid or ensemble models combine complementary strengths. For instance, GNN to capture 2D/3D molecular structures, recurrent architectures to handle sequential representations such as SMILES, and transformer-based models for long-range contextual dependencies [87]. These integrative approaches underscore the value of multi-perspective feature extraction over relying on a single model. Consequently, future benchmarking should evaluate not only raw performance but also the complementary strengths of different models.

**Table 4:** Comparison of deep learning models.

Model	Core Mechanism	Advantages	Disadvantages	Drug Discovery Applications
**CNNs**	Convolution-pooling stack [88]	Convolutional layers extract local features; pooling layers reduce dimensionality [89]	Relies on local receptive fields; struggles with global dependencies	Cellular morphology analysis, drug–drug interaction prediction [90]
**RNNs**	Recurrent connections	Process sequential data via cyclic connections [73,91,92,93]	Gradient vanishing/explosion; weak long-range dependency handling	SMILES-based molecular generation [94]
**GNNs**	Graph convolution	Aggregate neighborhood information through graph message-passing [95,96,97,98,99,100,101]	High computational complexity; hyperparameter sensitivity	Solubility prediction, binding affinity calculation [51,102,103]
**Transformers**	Self-attention mechanism [104]	Capture global dependencies via self-attention mechanisms [105,106]	High memory consumption; prone to overfitting on small datasets	Derivative design, multimodal data integration [69,107]
**GANs**	Generator-discriminator adversarial	Adversarial training between generator and discriminator	Training instability, mode collapse risk	Novel chemical entity synthesis [71]
**VAEs**	Encoder–decoder latent space	Compress molecular features via encoder–decoder architecture	Blurry generated samples, limited diversity	Targeted drug design [78]
**LLMs**	Multi-layer Transformer [108,109]	Multimodal Transformer-based joint reasoning	High training cost, requires domain-specific fine-tuning	Literature knowledge-driven molecular optimization [110]

Convolutional neural networks, CNNs. Recurrent neural networks, RNNs. Graph neural networks, GNNs. Generative adversarial networks, GANs. Variational autoencoders, VAEs. Large language models, LLMs.

## 3. Applications in New Drug Development

The rapid expansion of bioactive compounds and biomedical multi-omics data, accelerated by technologies such as high-throughput virtual screening, has necessitated advanced computational approaches in drug discovery. AI plays a key role in multiple drug development stages, including molecular characterization, structural analysis of target proteins, and virtual screening of lead compounds. ML algorithms, with their predictive modeling capabilities, continuously optimize the entire drug design process. The following sections will highlight innovative applications of AI in these areas, illustrating how technological breakthroughs are driving further advances in new drug development.

### 3.1. Applications in Tertiary Structure of Target Proteins

The tertiary structure of a protein is formed by the folding of its amino acid sequence through complex atomic interactions, directly determining its biological function [111]. Since protein dysfunction is a key pathological mechanism in many diseases, accurately determining its three-dimensional structure is critical for structure-based drug discovery [112,113]. However, the immense computational cost of modeling complex molecular folding and dynamics renders traditional simulation-based methods a bottleneck in early drug development.

AlphaFold has revolutionized the field of structural biology by enabling data-driven prediction of protein conformations directly from sequence information [114,115]. AlphaFold2 achieves remarkable accuracy in predicting monomeric protein folds but is inherently limited to static conformations. It does not capture dynamic processes, such as allosteric transitions, conformational flexibility, or ligand-induced rearrangements. Consequently, its application in protein–ligand and protein–protein interaction studies remain constrained. On the other hand, more recent frameworks like AlphaFold-Multimer and AlphaFold3 can predict complexes of proteins, nucleic acids, and small molecules. However, they still cannot fully model dynamic interactions, which remain dependent on molecular dynamics simulations [116,117]. Despite these limitations, such models have shown practical value in structure-based drug discovery. For instance, through computational prioritization and preclinical validation, researchers successfully identified a potent lead compound that acts as a trace amine-associated receptor 1 (TAAR1) agonist [118]. Moreover, integration of structure prediction with generative AI systems further empowers de novo drug design. For example, the PCMol model enables simultaneous multi-target candidate molecule optimization while maintaining synthetic feasibility [119]. In summary, the synergy between structure prediction and AI-driven chemistry platforms can be directly applied to hit identification in drug discovery, offering viable development pathways for understudied targets [120].

Beyond AlphaFold, several other DL frameworks contribute to tertiary structure prediction and protein design. The RoseTTAFold model can design entirely novel proteins not observed in nature, expanding the druggable structural space [121,122]. Similarly, the trRosetta (transform-restrained Rosetta) server is a deep learning-driven platform for rapid and accurate de novo protein structure prediction. Unlike traditional homology modeling approaches, trRosetta excels in de novo prediction when no experimentally resolved templates are available [123]. Meanwhile, ESMFold, a Transformer-based protein language model, achieves rapid sequence to structure prediction through training at various scales. It is up to ten times faster than traditional methods and maintains accuracy even for understudied proteins with limited structural data [113]. Although its precision is slightly below mainstream benchmarks, this speed advantage makes such models critical for streamlining early-stage drug design. Overall, these technological advancements are accelerating structure-guided therapeutic development by enhancing computational efficiency and enabling structural innovation.

### 3.2. Applications in Target Identification

In precision medicine, the growing complexity of multifactorial diseases has created an urgent need for new molecular targets. However, the number of clinically validated candidate drug targets is still limited, patient applicability is narrow, and clinical-trial failure rates remain high [124]. Until 2022, fewer than 500 drug targets had been successfully established worldwide, a figure that falls far short of the needs for complex disease treatment [125,126]. Consequently, discovering and evaluating new therapeutic targets has become a central focus of drug development. AI has emerged as a key enabler in this area by systematically integrating multi-scale biomedical data to perform probabilistic target identification with enhanced mechanistic interpretability [14].

Modern target discovery relies on three core strategies: experimental validation, multi-omics integration, and computational modeling. Experimental approaches use functional genomics tools like CRISPR-based screens to directly validate connections between targets and diseases [127,128,129,130,131]. Multi-omics platforms integrate genomic, proteomic, and metabolomic data to systematically identify how genes influence disease characteristics [132,133,134,135,136,137]. Computational methods use ML and structural biology to prioritize drug targets. Techniques like inverse docking (which screens a single ligand against multiple protein targets) and pharmacophore analysis help identify promising candidates, reducing the need for experimental resources [138,139,140].

**Figure 3:**
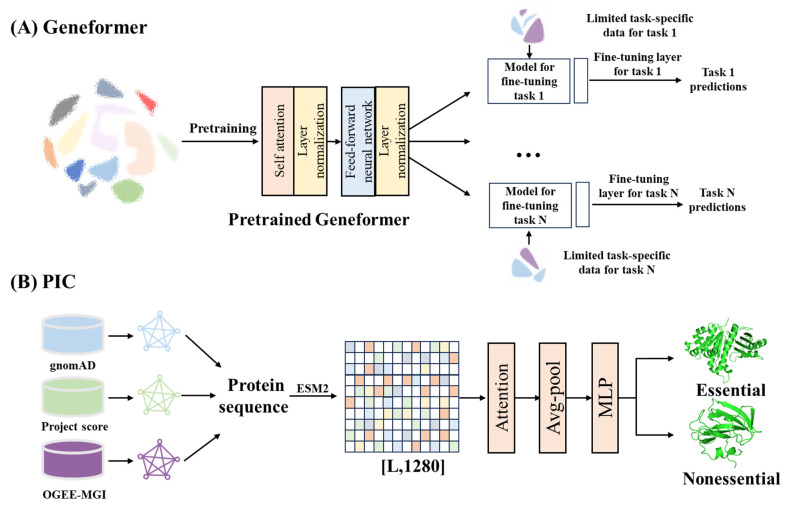
Applications of AI in target discovery. (**A**), Transformer-based gene network model for single-cell transcriptomics [142]. (**B**), Protein essentiality prediction framework integrating multi-source biological data [143]. ESM2, Evolutionary scale modeling 2. MLP, Multilayer perceptron. Avg-pool, Average pooling. Attention, Attention mechanism.

As biomedical datasets have grown, AI techniques leveraging large scale data analysis, pattern recognition and network construction have found broad application in target discovery. For example, AI analysis of transcriptome profiles from young and aged skeletal muscle identified key drivers of muscle aging [141]. At the same time, Transformer-based architectures have been particularly impactful for integrating multi-omics datasets. In single-cell transcriptomics, pre-trained transformer models learn gene relationships through attention mechanisms to capture gene–gene interactions. This enables researchers to accelerate the discovery of key network regulators and candidate therapeutic targets (Figure 3A) [142]. In parallel, building on pretrained protein language models have transformed target prioritization by integrating data across human, murine, and cellular contexts. Frameworks such as protein essentiality predictors use a multi-module structure that encodes protein sequences, weights biological context information, and classifies essentiality probabilities. These models generate protein essentiality scores that assist in identifying key therapeutic targets, cancer prognostic markers, and microprotein functions (Figure 3B) [143].

Alongside these strategies, by applying RefMap to human induced pluripotent stem cells (iPSC) derived motor neurons, the researchers mapped noncoding regulatory regions to target genes, identifying 690 ALS-associated candidates and achieving a fivefold increase in recovered heritability [144]. Finally, AI-powered drug-repurposing platforms (such as deepDTnet, which embeds chemical, genomic, phenotypic, and cellular network data) have successfully predicted topotecan as a related orphan receptor-gamma t inhibitor and demonstrated its efficacy in a mouse model of multiple sclerosis [145].

As algorithmic sophistication converges with expanding biomedical datasets, AI-driven target discovery is transitioning from exploratory research to clinical implementation. Through their bridging of mechanistic insights and therapeutic design, these technologies promise to streamline drug development cycles and advance precision medicine.

### 3.3. Applications in Drug–Target Interaction Prediction

Accurate prediction of drug–target interactions is a cornerstone of modern drug discovery, providing key insights to understand binding mechanisms and directly guiding rational therapeutic design. Molecular docking is a core computational approach in this field, aiming to predict the preferred orientation and conformation of small-molecule ligands when bound to protein targets, while estimating their binding affinity through scoring functions. By combining search algorithms and energy-based scoring, docking enables virtual screening of large chemical libraries to identify potential binders efficiently [146]. Thanks to the close integration of ML and structural biology, this field has made significant progress in recent years. One of the prominent directions is the innovation of virtual screening methods. For example, molecular docking approaches optimized through active learning employ intelligent sampling strategies to effectively balance chemical space exploration with the identification of potentially highly active molecules. Such methods have demonstrated the ability to recover over 80% of experimentally confirmed active compounds while reducing computational costs by a factor of 14. Moreover, over 90% of active scaffolds were retained within the top 5% of model-predicted compounds, preserving the diversity of confirmed actives [147].

Innovative techniques for drug–target interaction (DTI) prediction are overcoming traditional limitations. To address the challenge of limited training data and poor generalization, ML based on pre-trained protein language models have significantly enhanced extrapolation capabilities by analyzing the structural determinants of molecular recognition. These systems have been shown to accurately distinguish true binders from decoy compounds. Furthermore, they offer interpretable visualizations of interactions, enabling the use of embeddings to represent the functional characteristics of human cell surface proteins [148].

**Figure 4:**
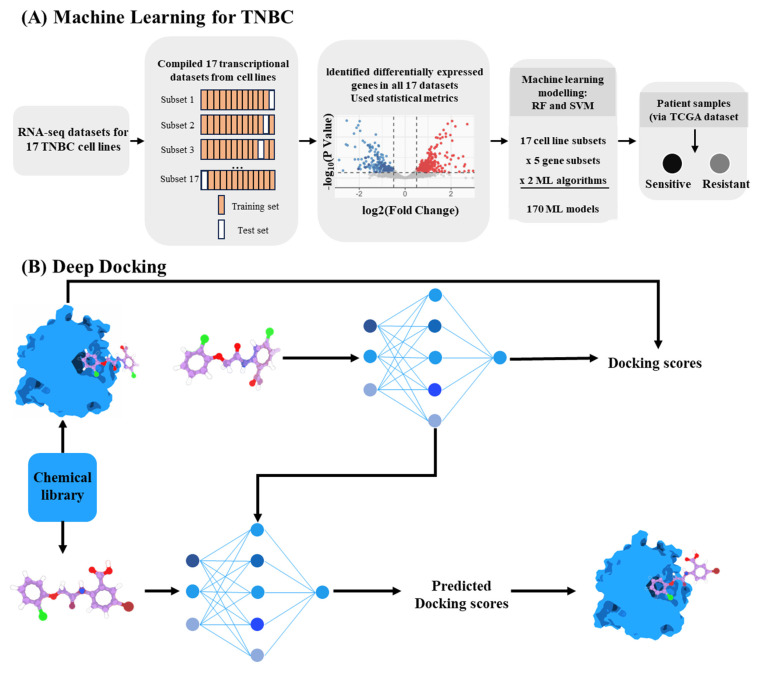
AI applications in drug–target interaction prediction. (**A**), Transcriptome-guided framework for target prioritization [149]. (**B**), Deep Docking approach combining molecular docking and neural prediction [150].

The deep integration of DL and molecular docking has catalyzed a transformation in virtual screening platforms. These platforms have been extended to multi-target pharmacological studies and to the creation of customized, synergistic kinase inhibition regimens for aggressive cancers through transcriptome-driven models (Figure 4A) [149]. Traditional docking involves generating candidate poses and scoring them based on binding energy. DL now learns from docking outcomes to accelerate screening across vast chemical libraries. For instance, Deep Docking utilizes QSAR deep models to estimate the docking outcome for yet unprocessed entries. In this approach, a small subset of compounds is first docked. A deep neural network is then trained on this subset to predict the docking scores for the remaining library, which dramatically reduces computation time while maintaining high accuracy (Figure 4B) [150,151]. Similarly, receptor activation pattern analysis could guide the development of dual-target agonists with enhanced pharmacological properties [152].

However, systemic challenges still need to be overcome. Rare diseases and emerging pathogens often lack sufficient labeled data, limiting model generalizability. Current predictors often suffer from reduced reliability due to dataset bias. To mitigate these issues, transfer learning and zero-shot learning strategies have been increasingly adopted. For example, TxGNN, a graph neural network model developed for zero-shot drug repurposing, leverages large-scale medical knowledge graphs to predict indications and contraindications even for diseases without existing therapies [95]. Future advances will likely require systematic curation of balanced interaction datasets combined with bias-resistant and transfer-learning-enabled modeling architectures to improve predictive reliability. Recent DL models in the target prediction tasks were collected in Table 5.

### 3.4. Applications in Virtual Screening of Lead Compounds

**Figure 5:**
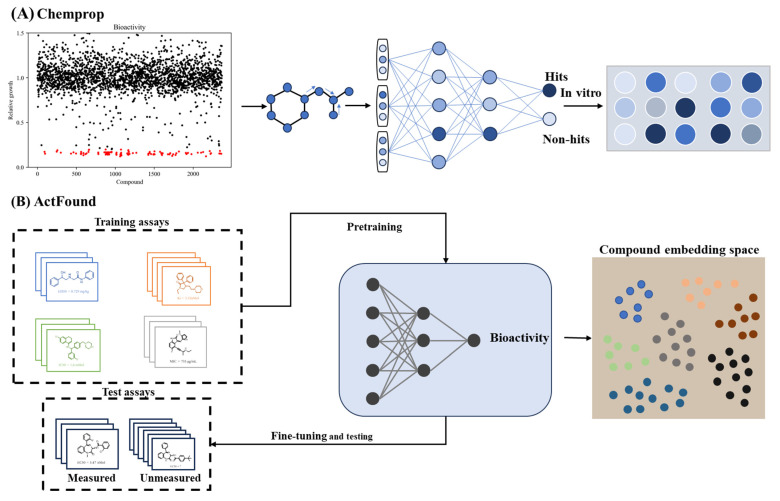
AI applications in lead compound virtual screening. (**A**), Graph-based neural network architecture for antimicrobial discovery [51]. (**B**), Meta-learning framework addressing data scarcity across assays [166].

Virtual screening has emerged as a transformative approach in lead compound identification, overcoming the cost and scalability limitations of traditional high-throughput screening (HTS). This capability is achieved through the AI-driven prioritization of bioactive molecules from extensive chemical libraries [161]. The integration of structural informatics with DL has proven particularly impactful in antimicrobial discovery. For instance, directed message-passing neural network (D-MPNN) captures complex atomic relationships by transmitting messages along chemical bonds, generating molecular embeddings that encode both local and global structural information. This approach achieves high predictive accuracy for antimicrobial and anticancer lead discovery (Figure 5A) [51]. This computational framework has been further extended to develop narrow-spectrum therapeutics selectively targeting high-risk pathogens like *Acinetobacter baumannii*, demonstrating precision in addressing antimicrobial resistance [162]. Subsequent innovations combining structural feature analysis with bioactivity prediction enabled the discovery of *Helicobacter pylori* growth inhibitors [163]. Despite these advances, the limited interpretability of DL remains a critical challenge in virtual screening. Recent explainable AI frameworks address this gap by incorporating chemical substructure recognition and structural analysis into graph-based neural networks, thereby maintaining interpretable design principles while exploring novel molecular scaffolds [28].

The therapeutic applications of such models span multiple disease domains, with notable progress in anticancer and metabolic drug development. ML approaches combining molecular feature encoding with dimensionality reduction techniques have identified validated antidiabetic natural [164]. In oncology, proteomics-driven prediction frameworks that integrate protein interaction networks demonstrate robust performance through noise-resistant algorithms. However, while these approaches show promise, their broader adoption requires addressing dataset limitations to mitigate overfitting risks inherent to proteomic-based deep learning models [165].

Finally, bioactivity prediction has been revolutionized through meta-learning architectures that mitigate data scarcity and experimental variability. A representative framework trained on cross-platform bioactivity data achieves robust generalization capabilities, enabling reliable predictions for novel drug development even with limited training samples (Figure 5B) [166]. Recent DL models in the virtual screening and bioactivity prediction tasks were collected in Table 6.

**Table 6:** DL models for virtual screening and bioactivity prediction.

No.	Model Name	Framework Description	Web
1	Chemprop	A deep learning package implementing Directed Message Passing Neural Networks (D-MPNNs) for molecular property prediction. It efficiently predicts physicochemical properties (such as logP, reaction barriers) and bioactivity, enabling rapid ADME/efficacy assessment in lead-compound screening to guide candidate optimization and reduce experimental cost [102].	https://github.com/chemprop/chemprop (accessed on 20 August 2025)
2	iPADD	Screening molecular fingerprint features through feature selection strategies to predict the activity of anti-diabetic compounds using an XGBoost model [164].	https://github.com/llllxw/iPADD/blob/main/README.md (accessed on 20 August 2025)
3	DRUMLR	An ensemble machine learning framework leverages proteomic and phosphoproteomic data to rank over 400 anticancer drugs by efficacy, enabling rapid prioritization of high-potential leads [165].	https://github.com/CutillasLab/DRUMLR (accessed on 20 August 2025)
4	ActFound	A meta-learning and pairwise-learning bioactivity model that rapidly adapts to small assay datasets to predict relative activity differences with high precision, requiring minimal fine-tuning [166].	https://github.com/BFeng14/ActFound (accessed on 20 August 2025)
5	TOML-BERT	This dual-level pretrained model combines self-supervised learning on molecular structures with domain-knowledge transfer using pseudo-labels. It integrates atomic and molecular-level tasks to achieve state-of-the-art ADMET prediction accuracy across ten drug datasets, especially when labeled data is scarc [167].	https://github.com/yanjing-duan/TOML-BERT (accessed on 20 August 2025)
6	FP-GNN	A hybrid GNN model that fuses molecular-graph structural information with fingerprint-based substructure features, markedly improving prediction accuracy for molecular properties [168].	https://github.com/idrugLab/FP-GNN (accessed on 20 August 2025)
7	ChemBERTa2	A model pretrained on chemical molecular structures (SMILES) that efficiently predicts a wide range of physicochemical and bioactivity properties [169].	https://github.com/miservilla/ChemBERTa (accessed on 20 August 2025)
8	Uni-Mol	This 3D molecular deep-learning framework is pretrained on extensive datasets of small molecules and protein binding pockets. It can directly predict molecular physicochemical properties, generate accurate 3D conformations, and simulate drug-target binding modes [170].	https://github.com/deepmodeling/Uni-Mol/tree/main/unimol (accessed on 20 August 2025)
9	SPMM	A multimodal molecular model that jointly learns from both structural representations and associated properties, enabling bidirectional prediction and generation [171].	https://github.com/jinhojsk515/SPMM/ (accessed on 20 August 2025)
10	MoLFormer	An efficient Transformer model pretrained on large-scale chemical SMILES datasets, capable of accurately predicting molecular properties to aid both drug discovery and materials design [172].	https://github.com/IBM/molformer (accessed on 20 August 2025)

TOML-BERT, Task-oriented multilevel learning based on BERT. FP-GNN, fingerprints and graph neural network. SPMM, Structure–Property Multi-Modal foundation model.

### 3.5. Application in the Generation of Lead Compounds

The identification and generation of lead compounds remain pivotal challenges in early drug discovery. Current estimates suggest the chemical space contains 10^23^–10^60^ potential drug-like molecules, yet only ~10^8^ have been synthesized to date [173,174]. Lead compound generation and optimization constitute one of the most critical bottlenecks in early-stage drug discovery, since even subtle structural modifications can profoundly influence potency, selectivity, pharmacokinetics, and safety profiles [175]. Traditional approaches such as molecular docking, and free-energy perturbation (FEP) have provided valuable guidance but often rely on predefined chemical rules or approximated scoring functions, limiting their capacity to explore the vast chemical space and to capture complex structure–activity relationships [176].

Deep generative models trained on structural patterns and bioactivity relationships enable de novo design of novel molecular entities with optimized drug-like properties, effectively expanding the scope of virtual screening libraries. Equivariant diffusion models enable the generation of novel ligands conditioned on protein pockets [177]. Meanwhile, toxicity-controlled ligand generation frameworks integrate safety considerations directly into the design process [178]. Additionally, DiffGui facilitates the simultaneous generation of atoms and bonds, producing molecules that exhibit high structural rationality and desirable molecular properties [179]. Compared with classical QSAR or docking-based strategies, these models not only enhance hit rates and novelty but also improve synthetic feasibility and safety awareness, thereby redefining the state of the art in lead optimization.

**Figure 6:**
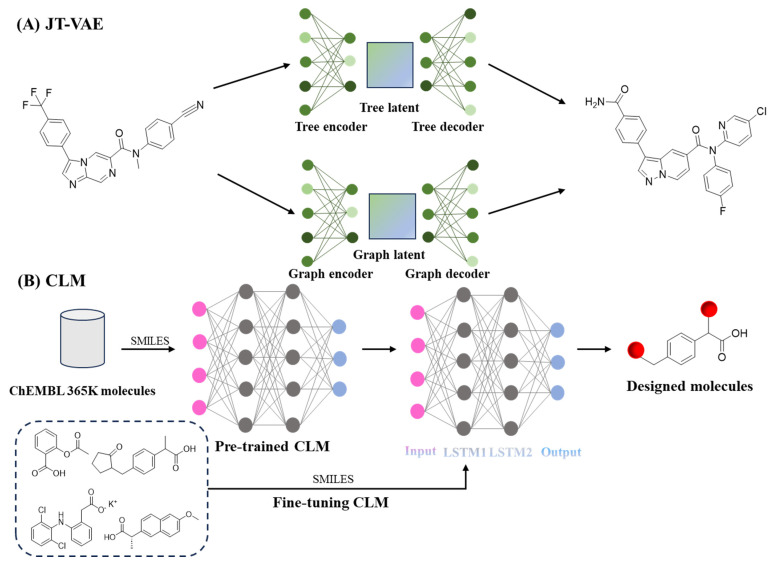
Application of AI in lead compound generation. (**A**), Variational autoencoder-based generative framework [180]. (**B**), Chemical language model (CLM) for SMILES-based molecular design [94]. LSTM, long short-term memory.

VAEs encode molecules into a latent space and reconstruct new structures while optimizing predicted bioactivity. This approach has successfully developed novel compounds with high potency against parasitic enzymes, exhibiting IC_50_ values of 0.023 μM and 0.025 μM against *P. falciparum* 3D7 (Figure 6A) [180]. The SyntheMol platform, through combining a validated library of chemical transformations with Monte Carlo tree search, generated structurally novel antibiotics with strong activity against multidrug-resistant *Acinetobacter baumannii* [181].For tuberculosis treatment, a target-aware generative system successfully identified protease inhibitors with measurable efficacy against *Mycobacterium tuberculosis*, discovering 14 compounds with significant inhibitory activity against the ClpP protease of *Mycobacterium tuberculosis*. The most potent compound had an IC_50_ of 1.9 μM [182].

Recent advances in chemical language modeling have further enhanced generative capabilities [183,184,185]. Chemical language models (CLMs), inspired by natural language processing, treat SMILES strings as sequences of tokens. Pretrained on large chemical datasets, these models learn the “grammar” of chemistry and can generate valid, synthetically accessible molecules. When fine-tuned on small datasets, CLMs can design dual-target ligands, such as multitarget compounds for metabolic disorders (Figure 6B) [94]. In a complementary approach, transcriptomic signature-driven generative models are designed to reconstruct drug-induced biological profiles. For example, they have been applied to repurpose therapies for pancreatic cancer by efficiently screening clinical compound libraries [186]. These synergistic innovations establish generative AI as a versatile platform for chemical space exploration. Their integration into high-throughput experimental workflows is accelerating therapeutic development across infectious diseases, metabolic disorders, and oncology. A summary of recent deep learning models for lead compound generation is provided in Table 7.

### 3.6. Applications in Synthesis Prediction in Drug Discovery

The integration of computational synthesis prediction into drug discovery pipelines has emerged as a transformative strategy for addressing the inherent complexity and cost challenges in therapeutic development [201]. Researchers embed AI within the design-make-test-analyze (DMTA) cycle to accelerate synthetic route planning, while also ensuring practical feasibility through reaction condition optimization and outcome validation [23]. For instance, Thakkar et al. employ a Monte Carlo tree search guided by learned policies to break down target molecules into purchasable precursors, typically completing full routes in under one minute. Building on that approach, a lightweight classifier predicts retrosynthetic accessibility thousands of times faster than a full search by estimating whether a viable route exists [202].

**Figure 7:**
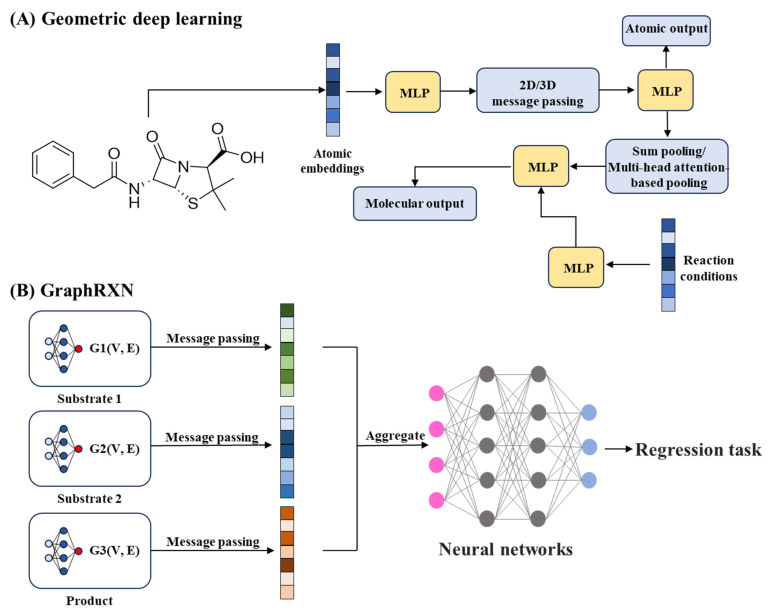
Applications of AI in chemical synthesis. (**A**), Geometric deep learning for regioselectivity prediction and late-stage functionalization [203]. (**B**), Graph-based and transformer models for reaction and yield prediction [205]. MLP, Multilayer perceptron.

Beyond route finding, predicting synthesis feasibility via learned metrics has matured significantly. DeepSA, trained on 3.6 million SMILES from public and proprietary sources, achieves an AUROC of 0.896 for synthesis accessibility. This model enables chemists to prioritize cost-effective and readily synthesizable molecules, reducing experimental attrition and accelerating lead optimization [22].AI-driven advances in reaction prediction prioritize spatial molecular interactions. In late-stage functionalization, where selective modification of complex molecular scaffolds is required, geometric deep learning plays a crucial role. With researchers integrating 3D atomic coordinates into GNNs, these models quantify both steric and electronic interactions to predict regioselectivity and reaction outcomes. This enables chemists to design site-selective transformations with improved efficiency and precision (Figure 7A) [203].

Complementary innovations address forward reaction prediction. Transformer-based models (e.g., T5Chem) treat reactions as text-to-text problems, achieving state-of-the-art accuracy in classification, retrosynthesis, and yield estimation on USPTO_500_MT data (SHAP analysis providing functional group-level interpretability) [204]. Graph-based reaction prediction systems, such as GraphRXN, represent another major innovation. Instead of relying on molecular descriptors, they directly encode reactant, reagent, and product graphs, node and edge features to learn reaction patterns. This end-to-end learning enables real-time product yield and regioselectivity prediction and allows seamless integration with robotic synthesis platforms, supporting fully automated chemical workflows (Figure 7B) [205].

With the maturation of multimodal data integration, AI-powered synthesis prediction stands poised to redefine pharmaceutical chemistry, transforming it into a fully data-driven discipline capable of intelligent molecular design and automated synthetic planning. Recent DL models in synthesis prediction tasks are collected in Table 8.

**Table 8:** DL Models for the synthesis prediction tasks.

No.	Model Name	Framework Description	Web
1	GSETransformer	An integrated model that combines graph neural networks and sequence processing to predict biosynthetic pathways of natural products. It accelerates route design in drug development and provides a visual interface that supports research workflow efficiency [206].	https://github.com/momozhangcn/GSETRetro (accessed on 20 August 2025)
2	Molecular Transformer	An attention-based model that unifies reaction prediction and retrosynthetic analysis for drug molecules. It maintains strong accuracy on novel compounds and serves as an effective tool for planning pharmaceutical syntheses [207].	https://github.com/pschwllr/MolecularTransformer (accessed on 20 August 2025)
3	DeepSA	A deep-learning chemical language model that predicts synthetic accessibility from molecular structure. It prioritizes easily synthesizable compounds and reduces both development time and cost [22].	https://github.com/Shihang-Wang-58/DeepSA (accessed on 20 August 2025)
4	Retro-MTGR	A multitask learning framework that uses molecular structure features to predict key bond disconnections and leaving groups in single-step retrosynthesis. It provides an efficient tool for planning synthetic routes [208].	https://github.com/zpczaizheli/Retro-MTGR (accessed on 20 August 2025)
5	LocalRetro	A retrosynthesis prediction model that integrates local molecular structure analysis with global attention mechanisms. It accurately designs synthetic routes for a broad range of drug-like molecules [209].	https://github.com/kaist-amsg/LocalRetro (accessed on 20 August 2025)
6	RetroTRAE	An atom-environment-aware model that predicts reactants directly for single-step retrosynthesis. It learns chemical fragment patterns and achieves 61.6% accuracy on benchmark datasets, surpassing SMILES-based methods [210].	https://github.com/knu-lcbc/RetroTRAE (accessed on 20 August 2025)
7	ReroSub	An end-to-end retrosynthesis model that automatically identifies conserved molecular substructures to simplify reaction prediction. It improves accuracy by over 5% compared with template-based approaches and removes the need for predefined reaction templates [211].	https://github.com/fangleigit/RetroSub (accessed on 20 August 2025)
8	G2GT	A hybrid framework combining graph networks and Transformer architectures in an encoder–decoder design. It uses self-training to predict required reactants for a target molecule and guides retrosynthetic route construction with high precision [212].	https://github.com/ZaiyunLin/G2GT_2 (accessed on 20 August 2025)
9	MolecularGET	A fusion model that combines graph neural networks with Transformer encoders to integrate structural and sequential chemical information. It enhances retrosynthesis prediction accuracy and accelerates route design [213].	https://github.com/papercodekl/MolecularGET (accessed on 20 August 2025)
10	Graph2SMILES	A template-free neural model that predicts reactants or products directly from molecular graphs. It improves the accuracy of both retrosynthesis and forward reaction prediction without complex preprocessing [214].	https://github.com/coleygroup/Graph2SMILES (accessed on 20 August 2025)
11	Graph2Edits	An edit-based architecture that applies stepwise graph modifications to infer feasible reactants from products. It achieves 55.1% accuracy in single-step prediction and performs well in complex multi-center transformations [208].	https://github.com/Jamson-Zhong/Graph2Edits (accessed on 20 August 2025)
12	RetroPrime	A two-stage Transformer model that first decomposes a target molecule and then generates plausible reactant sets. It improves retrosynthesis accuracy while increasing diversity and chemical feasibility of predicted routes [215].	https://github.com/wangxr0526/RetroPrime (accessed on 20 August 2025)
13	RAscore	A machine learning classifier that estimates synthetic accessibility scores for molecules. It enables large-scale prescreening to enrich chemical libraries with easily synthesizable compounds [202].	https://github.com/reymond-group/Rascore (accessed on 20 August 2025)
14	T5Chem	A sequence-to-sequence model based on SMILES representation that unifies multiple chemistry tasks, including reaction prediction, retrosynthesis, classification, and yield estimation. It also supports model interpretability studies [204].	https://github.com/HelloJocelynLu/t5chem (accessed on 20 August 2025)
15	GraphRXN	A graph neural network framework that analyzes 2D molecular structures of reactants and products to predict reaction outcomes. It demonstrates strong performance when trained with high-throughput experimental data [205].	https://github.com/jidushanbojue/GraphRXN (accessed on 20 August 2025)
16	Geometric deep learning	A 3D-geometry-based framework that integrates structural information with high-throughput experimentation to predict late-stage functionalization outcomes such as yield and regioselectivity. It accelerates physicochemical property optimization for complex drug candidates [203].	https://github.com/ETHmodlab/lsfml (accessed on 20 August 2025)

GSETransformer, graph-sequence enhanced transformer. Retro-MTGR, a Multi-Task Graph Representation learning framework for Retrosynthesis prediction. LocalRetro, a local retrosynthesis framework. G2T2, a graph-to-graph transformation model. Molecular GET, molecular graph enhanced transformer. RAscore, retrosynthetic accessibility score. T5Chem, “Text-to-Text Transfer Transformer” (T5) framework.

### 3.7. Absorption, Distribution, Metabolism, Excretion, and Toxicity (ADMET) Prediction in Drug Discovery

**Figure 8:**
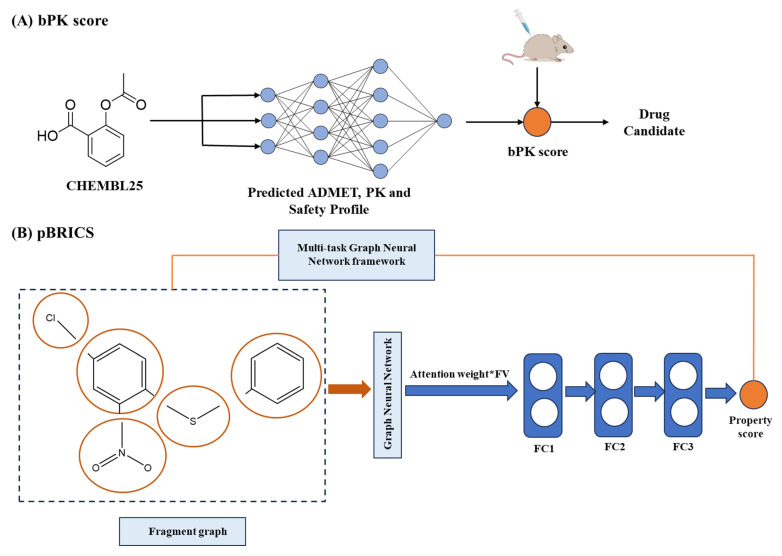
Applications of AI in ADMET. (**A**), Bayesian pharmacokinetic (bPK) framework integrating multi-descriptor data for compound prioritization [216]. (**B**), Fragment-based explainable model linking molecular substructures to pharmacokinetic properties [217]. FV, feature vector. FC, fully connected layers.

The accurate prediction of ADMET properties has become indispensable in modern drug discovery. AI has transformed this domain by enabling systematic analysis of molecular structure-pharmacokinetic relationships, overcoming the cost limitation of traditional experimental approaches. For instance, Bayesian pharmacokinetic (bPK) frameworks combine physicochemical and biological descriptors into composite metrics that quantitatively estimate molecular developability. These models outperform single-endpoint predictors by enabling multi-parameter optimization of absorption, bioavailability, and clearance profiles, effectively prioritizing compounds likely to succeed in downstream stages (Figure 8A) [216]. To address the interpretability–accuracy trade-off, fragment-based explainable AI (FBXAI) models have emerged. These methods decompose molecules into functional fragments and apply attention-based deep networks to learn how each substructure contributes to ADMET scores. Using domain-aware fragmentation and visualization tools such as Grad-CAM, the framework finds key functional domains responsible for properties like toxicity or metabolic stability (Figure 8B) [217].

Recent advances in toxicity prediction emphasize the integration of mechanistic interpretability with computational precision. Unified modeling approaches achieve superior discriminative power across diverse toxicity scores through DL-enhanced molecular descriptor analysis [218,219]. For example, specialized cardiac safety frameworks rigorously benchmark structural feature representations to elucidate drivers of multi-ion channel cardiotoxicity, significantly enhancing prediction reliability through structurally dissimilar validation sets [52]. Complementary platforms employ interpretable pattern recognition to decode structural determinants across diverse ADMET endpoints [220]. Furthermore, the field continues to evolve through large-scale open-source data initiatives that systematically address historical limitations in dataset diversity and balance, establishing comprehensive benchmarks for next-generation ADMET prediction [221]. Recent DL models in ADMET prediction tasks are collected in Table 9.

## 4. Future Challenges

Over the past decade, AI has driven transformative advances in drug discovery, particularly in compound property prediction and virtual screening, demonstrating its potential to shorten drug discovery cycles and reduce costs. However, AI is not a panacea for pharmaceutical R&D, as its integration faces multifaceted technical limitations, safety concerns, and ethical dilemmas that demand urgent resolution.

AI model performance is highly dependent on high-quality and sufficient datasets. Despite the vast scale of existing chemical libraries, rigorously curated datasets with robust biological, pharmacological, and clinical annotations remain scarce. This challenge is particularly acute in drug discovery, where the procurement of high-quality training data is hindered by extreme costs, stringent privacy regulations, and restrictive data-sharing agreements, especially for rare diseases and novel target studies [183]. Furthermore, biological variability poses a major obstacle to reproducibility and generalization. This variability, stemming from differences in experimental conditions, patient demographics, and genetic backgrounds, introduces significant noise and can lead to substantial fluctuations in molecular response data. Without careful normalization and multi-omics integration, AI models may learn these variations instead of the underlying biological signals, resulting in predictions that are biased, non-reproducible, and poorly generalizable to new populations or experimental settings.

Another critical issue is the lack of negative and failed experimental data in public databases. Because most published datasets overrepresent successful experiments, AI models are often unable to learn from failure modes, which are crucial for predicting adverse effects, toxicity, and off-target interactions [228]. Therefore, future efforts should emphasize the systematic curation of both positive and negative results, along with the establishment of shared, standardized data infrastructures to enhance cross-study comparability and robustness [229].

The black-box nature of DL models represents another major barrier to their adoption in regulated biomedical environments [230]. While DL architectures such as convolutional and Transformer-based networks have significantly advanced molecular representation learning, their lack of interpretability remains a fundamental limitation [228]. In particular, large models are prone to high-risk modeling illusions due to the inherent bias of the attention mechanism that leads to the generation process. In addition, the black-box nature of large models makes it difficult to meet the interpretable requirements of vertical applications. Developing inherently explainable AI frameworks capable of elucidating structure-activity relationships and toxicity pathways is essential to bridge this gap. Recent advances in attention mechanisms and causal inference models offer promising pathways toward interpretable predictions that align with domain expertise [28].

Computational efficiency represents another major obstacle. Although modern graphics processing units (GPUs) have improved training performance, state-of-the-art deep learning architectures, particularly large Transformer models, demand massive computational and energy resources, creating both environmental and practical challenges [231]. To mitigate these costs, future research should prioritize green computing strategies. This includes developing lightweight yet high-performing architectures to reduce carbon footprints without sacrificing predictive power. Furthermore, decentralized frameworks like federated learning can enhance sustainability by minimizing redundant data transfers and enabling secure, collaborative model development across institutions without centralizing sensitive data.

In summary, while AI-driven drug discovery has achieved significant progress, its continued success depends on addressing data scarcity, biological variability, model interpretability, and sustainability challenges. With the ongoing evolution of algorithmic efficiency, interdisciplinary data integration, and open-source model development, AI holds great promise to fundamentally reshape drug discovery—making it faster, greener, and more reliable for global health advancement.

## Figures and Tables

**Table 2:** Comparison of machine learning models.

Comparison Dimension	Supervised Learning	Unsupervised Learning	Semi-Supervised Learning	Reinforcement Learning
**Data Requirements**	Fully labeled datasets (input–output pairs)	Unlabeled data	Small amount of labeled data coupled with a large amount of unlabeled data	Dynamic environment interaction (state-action-reward signals)
**Core Functions**	Classification, Regression	Clustering, Dimensionality reduction	Label expansion & data augmentation	Strategy optimization & sequential decision-making
**Typical Algorithms**	Support Vector Machine (SVM); Logistic Regression; Random Forest [53]	Principal Component Analysis (PCA); t-SNE; K-means [53,54]	Graph-based Semi-supervised Learning [56,57]	Q-learning; Policy Gradient [53,60]
**Advantages**	High prediction accuracy; Clear objective functions	Reveals intrinsic data structures; No labeling cost	Mitigates labeled data scarcity; Enhances model generalization	Adapts to dynamic environments; Optimizes long-term goals
**Disadvantages**	Relies on high-quality labels; High overfitting risk	Poor interpretability of results; Requires manual validation of clusters	High model complexity; Performance depends on initial label quality	Slow training convergence; High computational cost
**Drug Discovery Applications**	Molecular toxicity prediction; Activity classification [51,52]	Chemical space visualization; Compound library deduplication [55]	Drug–target interaction prediction [61]	Inhibitor reaction path optimization; Multi attribute molecular design [58,59]

**Table 3:** Comparative overview of commonly used shallow machine learning algorithms.

Model	Core Mechanism	Advantages	Disadvantages	Drug Discovery Applications
**Support Vector Machine (SVM)**	Finds optimal hyperplane maximizing the margin between classes using kernel functions.	High classification accuracy for small and high-dimensional datasets; robust to overfitting with suitable kernel.	Sensitive to kernel and hyperparameter selection; limited scalability for large datasets.	Drug–target interaction prediction; compound activity classification; ADMET and toxicity modeling.
**Decision Tree**	Builds hierarchical decision rules by recursively splitting features that maximize information gain.	Simple, interpretable, and requires minimal preprocessing; handles nonlinear relationships.	Prone to overfitting; small data perturbations can change structure.	Compound classification; QSAR modeling; structure–activity relationship visualization.
**Random Forest (RF)**	Ensemble of multiple decision trees via bootstrap aggregation (bagging) and majority voting.	Reduces variance and overfitting; robust to noise; provides feature importance ranking.	Decreased interpretability; slower inference for large forests.	Bioactivity prediction; virtual screening; toxicity classification.
**k-Nearest Neighbors (KNN)**	Classifies samples by majority vote of k nearest data points in feature space.	Simple implementation; adaptable to diverse datasets.	Computationally expensive for large datasets; sensitive to irrelevant features and scaling.	Molecular similarity searching; ligand-based virtual screening; QSAR/QSPR modeling.
**Artificial Neural Network (ANN)**	Composed of interconnected layers of neurons performing weighted summations and nonlinear activation.	Learns nonlinear mappings; flexible architecture; high predictive performance with proper tuning.	Prone to overfitting; requires large labeled data; limited interpretability compared to tree models.	QSAR/QSPR modeling; bioactivity prediction; pharmacokinetic property estimation.

**Table 5:** DL Models for target prediction frameworks.

No.	Model Name	Framework Description	Web
**1**	**Geneformer**	A deep learning model based on a six-layer Transformer architecture. It is self-supervised pretrained on 30 million single-cell transcriptomes to learn gene interaction dynamics. Its attention mechanisms autonomously capture hierarchical gene relationships, enabling transfer to data-scarce scenarios such as disease gene prediction, chromatin interaction analysis, and therapeutic target discovery [142].	https://huggingface.co/ctheodoris/Geneformer (accessed on 20 August 2025)
**2**	**PIC**	A framework built on the pretrained Evolutionary Scale Modeling 2 (ESM2) protein language model. It extracts sequence features via an embedding module, captures residue importance through an attention module, and outputs probability-based predictions of protein essentiality across human tissues, cell lines, and mouse. The resulting Protein Essentiality Score quantifies protein importance, supports breast-cancer prognostic marker identification, and evaluates microprotein function [143].	https://github.com/KangBoming/PIC (accessed on 20 August 2025)
**3**	**Deep Docking**	A binary classifier deep neural network trained iteratively on 1024-bit circular Morgan fingerprint features. It dynamically predicts and filters high-docking-score compounds from ultra-large libraries, reducing a multi-billion-member library to a manageable size while retaining over 90% of active candidates and drastically lowering compute requirements [150].	https://github.com/jamesgleave/DD_protocol (accessed on 20 August 2025)
**4**	**EMCIP**	An ensemble model combining multiple machine-learning algorithms with a multi-instance 3D graph neural network. Using stacked generalization and soft-voting, it integrates predictions from all base models for efficient forecasting of Candida drug resistance inhibitors [153].	https://github.com/trinhthechuong/Cdr1_inhibitors (accessed on 20 August 2025) https://huggingface.co/spaces/thechuongtrinh/EMCIP_Cdr1_inhibitor_prediction (accessed on 20 August 2025)
**5**	**CSNN**	A graph-neural network leveraging Chemical Space Networks (CSNs) and label information as node features. This method achieves zero-training predictions by querying a compound’s chemical neighbors and exploiting network homophily, significantly enhancing drug–target interaction accuracy for human G protein-coupled receptors [154].	https://github.com/cansyl/TransferLearning4DTI/ (accessed on 20 August 2025)
**6**	**GraphBAN**	A graph-based knowledge-distillation model combining a graph autoencoder and a bilinear attention network. It fuses multi-source features of compounds and proteins, along with a cross-domain adaptation module, to predict interactions between unseen nodes, improving both accuracy and generalization in compound–protein interaction prediction for drug discovery [155].	https://github.com/HamidHadipour/GraphBAN/blob/main/README.md (accessed on 20 August 2025)
**7**	**DTIAM**	A unified framework that uses self-supervised pretraining for drug molecules (multi-task learning) and Transformer-based protein representations. It incorporates automated machine-learning to predict drug–target interactions, binding affinities, and activation/inhibition mechanisms [156].	https://github.com/CSUBioGroup/DTIAM (accessed on 20 August 2025)
**8**	**KinGen**	A recurrent neural network (RNN)-based molecule generator combined with reinforcement learning and transfer learning. By optimizing reward functions and transferring knowledge, it could generate high-activity compounds targeting specific kinases [157].	https://github.com/Shawn-Lau-lxm/KinGen (accessed on 20 August 2025)
**9**	**MINDG**	A hybrid architecture that extracts drug and target sequence features via deep networks, captures higher-order structural information through graph-attention convolutional layers, and integrates multi-view predictions with an adaptive decision module. This approach markedly improves drug–target interaction prediction accuracy [158].	https://github.com/AGI-FBHC/MINDG (accessed on 20 August 2025)
**10**	**PIGNet2**	A DL model combining physics-informed graph neural networks with novel data-augmentation strategies. It accurately predicts protein–ligand binding affinities and efficiently screens candidate drugs, demonstrating exceptional cross-task performance in hit identification and lead optimization [159].	https://github.com/ACE-KAIST/PIGNet2 (accessed on 20 August 2025)
**11**	**CarsiDock**	A DL-based protein–ligand docking model pretrained on 9 million predicted complexes. Coupled with geometric optimization strategies, it greatly enhances pose-prediction accuracy, reliably reproduces key interactions observed in crystal structures, and preserves ligand topology for high-throughput virtual screening [160].	https://github.com/carbonsilicon-ai/CarsiDock (accessed on 20 August 2025)

PIC, Protein Importance Calculator. EMCIP, Ensemble Model for Cdr1 Inhibitor Prediction. CSNN, Chemical Space Neural Network. MINDG, Multi-view Integrated Learning Network.

**Table 7:** DL models for the generation of lead compounds.

No.	Model Name	Framework Description	Web
1	DRLinker	A deep reinforcement learning framework for fragment-based drug design that optimizes linker generation, controls physicochemical properties, enhances bioactivity, and promotes structural innovation during lead optimization [187].	https://github.com/biomed-AI/Drlinker (accessed on 20 August 2025)
2	Link-INVENT	Link-INVENT uses an RNN to generate novel linkers as SMILES strings between two molecular subunits. Unlike database-search methods, it creates linkers token-by-token, enabling exploration of new chemical space. Its key advantage is a customizable Scoring Function that uses reinforcement learning to optimize linkers for multiple specific properties, as demonstrated in fragment linking, scaffold hopping, and proteolysis targeting chimeras design case studies [188].	https://github.com/MolecularAI/Reinvent (accessed on 20 August 2025)
3	DeepHop	Integrates 3D molecular conformations and target protein information to efficiently generate compounds with novel 2D scaffolds, improved bioactivity, and preserved 3D similarity. It is specifically used for scaffold-hopping optimization in drug design [189].	https://github.com/prokia/deepHops (accessed on 20 August 2025)
4	ScaffoldGVAE	A deep generative model that smartly modifies the molecular core scaffold while retaining key side chains, enabling the design of novel active compounds. It has been successfully applied to develop Leucine-rich repeat ki-nase 2 inhibitors for Parkinson’s disease, offering a tool for innovative chemical-space exploration [75].	https://github.com/ecust-hc/ScaffoldGVAE (accessed on 20 August 2025)
5	MolGPT	A Transformer-based molecular generator that assembles chemically valid drug molecules and provides controllable optimization of structure and molecular properties [190].	https://github.com/devalab/molgpt (accessed on 20 August 2025)
6	Scaffold Decorator	A SMILES-based generative model that decorates a given scaffold to pro-duce novel compounds with potential activity. It has experimentally generated predicted Dopamine Receptor D2 ligands while ensuring synthetic feasibility, offering an effective scaffold-decoration tool [191].	https://github.com/undeadpixel/reinvent-scaffold-decorator (accessed on 20 August 2025)
7	SAMOA	A deep learning framework for lead optimization that maintains a specified core scaffold while improving bioactivity and drug-likeness under structural constraints [192].	https://github.com/maxime-langevin/scaffold-constrained-generation (accessed on 20 August 2025)
8	LibINVENT	A reaction-guided molecular generation platform that constructs libraries of compounds sharing a common scaffold, streamlining lead optimization and improving synthetic accessibility [193].	https://github.com/MolecularAI/Lib-INVENT (accessed on 20 August 2025)
9	DiffDec	A structure-aware molecule optimization tool based on diffusion models. Given a protein binding pocket’s 3D structure, it intelligently generates side-chain R-groups to enhance binding affinity, offering an efficient solution for lead optimization [194].	https://github.com/biomed-AI/DiffDec (accessed on 20 August 2025)
10	DeepFrag	An AI method using diffusion models that, guided by a protein’s 3D structure, generates chemically compatible R-groups for lead molecules, significantly boosting binding affinity and drug-likeness [195].	https://durrantlab.pitt.edu/deepfragmodel/ (accessed on 20 August 2025)
11	STRIFE	A structure-guided fragment expansion tool that designs optimized fragments based on target protein structures, improving efficiency for antiviral and anti-inflammatory drug discovery [196].	https://github.com/oxpig/STRIFE (accessed on 20 August 2025)
12	DEVELOP	A 3D-pharmacophore-guided generative framework (DEVELOP) that combines graph neural networks with 3D pharmacophoric features for linker and R-group design, improving target specificity and molecular quality [197].	https://github.com/oxpig/DEVELOP (accessed on 20 August 2025)
13	DrugEX-v3	A graph-transformer and reinforcement-learning model that generates high-affinity compounds from user-defined fragments [198].	https://github.com/CDDLeiden/DrugEx (accessed on 20 August 2025)
16	REINVENT4	A hybrid RNN/Transformer generative framework enhanced with reinforcement learning for de novo molecule generation, R-group replacement, linker design, and property optimization in an end-to-end workflow [199].	https://github.com/MolecularAI/REINVENT4 (accessed on 20 August 2025)
17	TamGen	A GPT-based chemical language model that generates new drug-like molecules targeting disease proteins and optimizes existing scaffolds for improved activity and synthetic accessibility [182].	https://github.com/SigmaGenX/TamGen/tree/main/data (accessed on 20 August 2025)
18	ClickGen	An AI model combining modular reaction chemistry with reinforcement learning to rapidly design easily synthesizable and bioactive drug molecules [200].	https://github.com/mywang1994/cligen_gen (accessed on 20 August 2025)
19	JAEGER	A deep generative model integrating structure generation with activity prediction, successfully designing low-toxicity antimalarials with nanomolar experimental efficacy [180].	https://github.com/Novartis/JAEGER (accessed on 20 August 2025)
20	SyntheMol	An AI–synthetic-chemistry hybrid system that rapidly proposes easily synthesizable novel antibiotic scaffolds. Experimentally, it identified six new compounds effective against drug-resistant Acinetobacter baumannii and other pathogens [181].	https://github.com/swansonk14/SyntheMol (accessed on 20 August 2025)
21	CLM	An RNN-based generative model trained on limited molecular data to produce structures containing specified motifs, applied to discover bacterial, plant, and fungal metabolites as potential drug leads [183].	https://github.com/skinnider/low-data-generative-models (accessed on 20 August 2025)

SAMOA, Scaffold constrained molecular generation. STRIFE, Structure informed fragment elaboration. DEVELOP, DEep Vision-Enhanced Lead OPtimisation. TamGen, Target-aware molecular generation. JAEGER, JT-VAE Generative Modeling. CLM, Chemical language model.

**Table 9:** DL Models for the ADMET prediction tasks.

No.	Model Name	Framework Description	Web
1	ADMET-PrInt	An online platform that integrates machine learning models with interpretability modules to predict major ADMET properties. It provides visual analytics of structural features to guide molecular optimization and accelerate in silico lead evaluation [218].	https://github.com/JamEwe/ADMET-PrInt (accessed on 20 August 2025)
2	PharmaBench	A benchmark dataset built from more than 14,000 bioassay entries covering over 52,000 compounds. It offers a standardized training and evaluation framework for AI models in absorption, distribution, metabolism, excretion, and toxicity prediction [221].	https://github.com/mindrank-ai/PharmaBench (accessed on 20 August 2025)
3	ADMET-AI	A hybrid platform combining graph neural networks and molecular descriptors for rapid ADMET property prediction. It supports both web and local deployment for high-throughput virtual screening focused on metabolism and toxicity endpoints [222].	https://admet.ai.greenstonebio.com/ (accessed on 20 August 2025)
4	SwissADME	A free web service that evaluates small-molecule ADME properties, drug-likeness, and synthetic accessibility. It assists researchers in early-stage drug candidate optimization [223].	http://www.swissadme.ch/ (accessed on 20 August 2025)
5	ADMETlab 3.0	A deep learning platform that predicts key ADMET parameters using integrated molecular features and neural network models. It streamlines early screening and reduces experimental workload [224].	https://admetlab3.scbdd.com/server/screening (accessed on 20 August 2025)
6	pkCSM	A graph-based predictive model that estimates metabolic stability and potential toxicity directly from molecular structure. It uses graph signatures to rapidly identify high-risk compounds during early ADMET assessment [225].	https://biosig.lab.uq.edu.au/pkcsm/ (accessed on 20 August 2025)
7	vNN-ADMET	A similarity-based prediction algorithm that applies nearest-neighbor statistics in chemical space. It provides ADMET predictions with confidence intervals to support risk-aware decision-making in candidate selection [226].	https://vnnadmet.bhsai.org/vnnadmet/login.xhtml (accessed on 20 August 2025)
8	ChemMORT	An automated optimization platform that combines reversible molecular representations with reinforcement learning. It refines ADME and safety properties while preserving bioactivity [227].	https://cadd.nscc-tj.cn/deploy/chemmort/ (accessed on 20 August 2025)
9	Conformal ADMET Prediction	A graph-neural-network model enhanced with conformal prediction calibration that generates both point estimates and prediction intervals for ADMET endpoints. It offers statistical confidence for decision-making under uncertainty [101].	https://github.com/peiyaoli/Conformal-ADMET-Prediction (accessed on 20 August 2025)

vNN-ADMET, variable nearest neighbor-ADMET. ChemMORT, Chemical Molecular Optimization, Representation and Translation.

## Data Availability

Not applicable. No new data were created or analyzed in this study. Data sharing is not applicable to this article.

## References

[1] Jung Y.L., Yoo H.S., Hwang J. (2022). Artificial intelligence-based decision support model for new drug development planning. Expert Syst. Appl..

[2] Wouters O.J., McKee M., Luyten J. (2020). Estimated Research and Development Investment Needed to Bring a New Medicine to Market, 2009–2018. JAMA.

[3] Harrison R.K. (2016). Phase II and phase III failures: 2013–2015. Nat. Rev. Drug Discov..

[4] Dowden H., Munro J. (2019). Trends in clinical success rates and therapeutic focus. Nat. Rev. Drug Discov..

[5] Smietana K., Siatkowski M., Møller M. (2016). Trends in clinical success rates. Nat. Rev. Drug Discov..

[6] Sun D., Gao W., Hu H., Zhou S. (2022). Why 90% of clinical drug development fails and how to improve it?. Acta Pharm. Sin. B.

[7] Biotechnology Innovation Organization, Informa Pharma Intelligence, Quantitative Life Sciences Advisors (2021). Clinical Development Success Rates and Contributing Factors 2011–2020. https://www.bio.org/clinical-development-success-rates-and-contributing-factors-2011-2020.

[8] Peng C., Zhao S., Tang L., Wang K., Wang Y., Ding L. (2020). A simplified and reliable LC-tandem mass spectrometry method for determination of ulipristal acetate in human plasma and its application to a pharmacokinetic study in healthy Chinese volunteers. Biomed. Chromatogr..

[9] Lyu J., Wang S., Balius T.E., Singh I., Levit A., Moroz Y.S., O’Meara M.J., Che T., Algaa E., Tolmachova K. (2019). Ultra-large library docking for discovering new chemotypes. Nature.

[10] Talukder M.E.K., Atif M.F., Siddiquee N.H., Rahman S., Rafi N.I., Israt S., Shahir N.F., Islam M.T., Samad A., Wani T.A. (2025). Molecular docking, QSAR, and simulation analyses of EGFR-targeting phytochemicals in non-small cell lung cancer. J. Mol. Struct..

[11] Kaur N., Gupta S., Pal J., Bansal Y., Bansal G. (2025). Design of BBB permeable BACE-1 inhibitor as potential drug candidate for Alzheimer disease: 2D-QSAR, molecular docking, ADMET, molecular dynamics, MMGBSA. Comput. Biol. Chem..

[12] Souza A.S., Amorim V.M.F., Soares E.P., de Souza R.F., Guzzo C.R. (2025). Antagonistic Trends Between Binding Affinity and Drug-Likeness in SARS-CoV-2 Mpro Inhibitors Revealed by Machine Learning. Viruses.

[13] Maliyakkal N., Kumar S., Bhowmik R., Vishwakarma H.C., Yadav P., Mathew B. (2025). Two-dimensional QSAR-driven virtual screening for potential therapeutics against *Trypanosoma cruzi*. Front. Chem..

[14] Pun F.W., Ozerov I.V., Zhavoronkov A. (2023). AI-powered therapeutic target discovery. Trends Pharmacol. Sci..

[15] Cassan O., Lèbre S., Martin A. (2021). Inferring and analyzing gene regulatory networks from multi-factorial expression data: A complete and interactive suite. BMC Genom..

[16] Nogales C., Mamdouh Z.M., List M., Kiel C., Casas A.I., Schmidt H.H.H.W. (2022). Network pharmacology: Curing causal mechanisms instead of treating symptoms. Trends Pharmacol. Sci..

[17] Zhuo C., Gao J., Li A., Liu X., Zhao Y. (2024). A Machine Learning Method for RNA–Small Molecule Binding Preference Prediction. J. Chem. Inf. Model..

[18] Zhou J.-B., Tang D., He L., Lin S., Lei J.H., Sun H., Xu X., Deng C.-X. (2023). Machine learning model for anti-cancer drug combinations: Analysis, prediction, and validation. Pharmacol. Res..

[19] He D., Liu Q., Mi Y., Meng Q., Xu L., Hou C., Wang J., Li N., Liu Y., Chai H. (2024). De Novo Generation and Identification of Novel Compounds with Drug Efficacy Based on Machine Learning. Adv. Sci..

[20] Huang L., Xu T., Yu Y., Zhao P., Chen X., Han J., Xie Z., Li H., Zhong W., Wong K.-C. (2024). A dual diffusion model enables 3D molecule generation and lead optimization based on target pockets. Nat. Commun..

[21] Zhou G., Rusnac D.-V., Park H., Canzani D., Nguyen H.M., Stewart L., Bush M.F., Nguyen P.T., Wulff H., Yarov-Yarovoy V. (2024). An artificial intelligence accelerated virtual screening platform for drug discovery. Nat. Commun..

[22] Wang S., Wang L., Li F., Bai F. (2023). DeepSA: A deep-learning driven predictor of compound synthesis accessibility. J. Cheminform..

[23] Struble T.J., Alvarez J.C., Brown S.P., Chytil M., Cisar J., DesJarlais R.L., Engkvist O., Frank S.A., Greve D.R., Griffin D.J. (2020). Current and Future Roles of Artificial Intelligence in Medicinal Chemistry Synthesis. J. Med. Chem..

[24] Ye Z., Wang N., Zhou J., Ouyang D. (2024). Organic crystal structure prediction via coupled generative adversarial networks and graph convolutional networks. Innovation.

[25] Yang Z., Zhao Y.-M., Wang X., Liu X., Zhang X., Li Y., Lv Q., Chen C.Y.-C., Shen L. (2024). Scalable crystal structure relaxation using an iteration-free deep generative model with uncertainty quantification. Nat. Commun..

[26] Ryan K., Lengyel J., Shatruk M. (2018). Crystal Structure Prediction via Deep Learning. J. Am. Chem. Soc..

[27] Ren F., Aliper A., Chen J., Zhao H., Rao S., Kuppe C., Ozerov I.V., Zhang M., Witte K., Kruse C. (2025). A small-molecule TNIK inhibitor targets fibrosis in preclinical and clinical models. Nat. Biotechnol..

[28] Wong F., Zheng E.J., Valeri J.A., Donghia N.M., Anahtar M.N., Omori S., Li A., Cubillos-Ruiz A., Krishnan A., Jin W. (2024). Discovery of a structural class of antibiotics with explainable deep learning. Nature.

[29] Nobel Prize in Chemistry 2024. https://www.nature.com/collections/edjcfdihdi.

[30] Van de Sande B., Lee J.S., Mutasa-Gottgens E., Naughton B., Bacon W., Manning J., Wang Y., Pollard J., Mendez M., Hill J. (2023). Applications of single-cell RNA sequencing in drug discovery and development. Nat. Rev. Drug Discov..

[31] Yang F., Wang W., Wang F., Fang Y., Tang D., Huang J., Lu H., Yao J. (2022). scBERT as a large-scale pretrained deep language model for cell type annotation of single-cell RNA-seq data. Nat. Mach. Intell..

[32] Chen J., Wang X., Ma A., Wang Q.-E., Liu B., Li L., Xu D., Ma Q. (2022). Deep transfer learning of cancer drug responses by integrating bulk and single-cell RNA-seq data. Nat. Commun..

[33] Hou R., Xie C., Gui Y., Li G., Li X. (2023). Machine-Learning-Based Data Analysis Method for Cell-Based Selection of DNA-Encoded Libraries. ACS Omega.

[34] Recursion Recursion’s Drug Pipeline. https://www.recursion.com/pipeline.

[35] Insilico Medicine Insilico Medicine’s Drug Pipeline. https://insilico.com/.

[36] Relay Therapeutics Relay Therapeutics’s Drug Pipeline. https://relaytx.com/pipeline/.

[37] Exscientia Exscientia’s Drug Pipeline. https://www.exscientia.com/pipeline/.

[38] Signet Therapeutics Signet Therapeutics’ Drug Pipeline. https://www.signettx.com/about/.

[39] BeiGene BeiGene’s Drug Pipeline. https://www.beonemedicines.com.cn/science/pipeline/.

[40] RedCloud Bio RedCloud Bio’s Drug Pipeline. http://www.redcloudbio.com/en/h-col-105.html.

[41] Accutar Biotech Accutar Biotech’s Drug Pipeline. https://www.accutarbio.com/workflow/.

[42] MindRank MindRank’s Drug Pipeline. https://www.mindrank.ai/zh-CN/pipeline.

[43] Drug Farm Drug Farm’s Drug Pipeline. https://www.drug-farm.com/pipeline.

[44] OrphAI Therapeutics OrphAI Therapeutics’s Drug Pipline. https://www.orphai-therapeutics.com/pipeline.

[45] Healx Healx’s Drug Pipeline. https://healx.ai/pipeline/.

[46] BioAge BioAge’s Drug Pipeline. https://bioagelabs.com/apj.

[47] BioXcel Therapeutics BioXcel Therapeutics’s Drug Pipeline. https://www.bioxceltherapeutics.com/our-pipeline/.

[48] Evaxion Biotech Evaxion Biotech’s Drug Pipeline. https://evaxion.ai/pipeline.

[49] Deo R.C. (2015). Machine Learning in Medicine. Circulation.

[50] Jiang T., Gradus J.L., Rosellini A.J. (2020). Supervised Machine Learning: A Brief Primer. Behav. Ther..

[51] Stokes J.M., Yang K., Swanson K., Jin W., Cubillos-Ruiz A., Donghia N.M., MacNair C.R., French S., Carfrae L.A., Bloom-Ackerman Z. (2020). A deep learning approach to antibiotic discovery. Cell.

[52] Arab I., Egghe K., Laukens K., Chen K., Barakat K., Bittremieux W. (2024). Benchmarking of Small Molecule Feature Representations for hERG, Nav1.5, and Cav1.2 Cardiotoxicity Prediction. J. Chem. Inf. Model..

[53] Singh S., Kaur N., Gehlot A. (2024). Application of artificial intelligence in drug design: A review. Comput. Biol. Med..

[54] Glielmo A., Husic B.E., Rodriguez A., Clementi C., Noé F., Laio A. (2021). Unsupervised Learning Methods for Molecular Simulation Data. Chem. Rev..

[55] Cihan Sorkun M., Mullaj D., Koelman J.M.V.A., Er S. (2022). ChemPlot, a Python Library for Chemical Space Visualization. Chem. –Methods.

[56] van Engelen J.E., Hoos H.H. (2020). A survey on semi-supervised learning. Mach. Learn..

[57] Niu Q., Li H., Tong L., Liu S., Zong W., Zhang S., Tian S., Wang J., Liu J., Li B. (2023). TCMFP: A novel herbal formula prediction method based on network target’s score integrated with semi-supervised learning genetic algorithms. Brief. Bioinform..

[58] Jiang X., Lu L., Li J., Jiang J., Zhang J., Zhou S., Wen H., Cai H., Luo X., Li Z. (2024). Synthetically Feasible De Novo Molecular Design of Leads Based on a Reinforcement Learning Model: AI-Assisted Discovery of an Anti-IBD Lead Targeting CXCR4. J. Med. Chem..

[59] Wang Y., Hu Z., Chang J., Yu B. (2025). Thinking on the Use of Artificial Intelligence in Drug Discovery. J. Med. Chem..

[60] Botvinick M., Ritter S., Wang J.X., Kurth-Nelson Z., Blundell C., Hassabis D. (2019). Reinforcement Learning, Fast and Slow. Trends Cogn. Sci..

[61] Xie Z., Tu S., Xu L. (2024). Multilevel Attention Network with Semi-supervised Domain Adaptation for Drug-Target Prediction. Proc. AAAI Conf. Artif. Intell..

[62] LeCun Y., Bengio Y., Hinton G. (2015). Deep learning. Nature.

[63] Kusumoto D., Seki T., Sawada H., Kunitomi A., Katsuki T., Kimura M., Ito S., Komuro J., Hashimoto H., Fukuda K. (2021). Anti-senescent drug screening by deep learning-based morphology senescence scoring. Nat. Commun..

[64] Grebner C., Matter H., Plowright A.T., Hessler G. (2020). Automated De Novo Design in Medicinal Chemistry: Which Types of Chemistry Does a Generative Neural Network Learn?. J. Med. Chem..

[65] Chen Y., Wang Z., Zeng X., Li Y., Li P., Ye X., Sakurai T. (2023). Molecular language models: RNNs or transformer?. Brief. Funct. Genom..

[66] Shor B., Schneidman-Duhovny D. (2024). DockFormer: Efficient Multi-Modal Receptor-Ligand Interaction Prediction using Pair Transformer. bioRxiv.

[67] Su X., Hu P., You Z.-H., Yu P.S., Hu L. (2024). Dual-Channel Learning Framework for Drug-Drug Interaction Prediction via Relation-Aware Heterogeneous Graph Transformer. Proc. AAAI Conf. Artif. Intell..

[68] Teng S., Yin C., Wang Y., Chen X., Yan Z., Cui L., Wei L. (2023). MolFPG: Multi-level fingerprint-based Graph Transformer for accurate and robust drug toxicity prediction. Comput. Biol. Med..

[69] Wei G.-W., Chen D., Liu J. (2024). TopoFormer: Multiscale Topology-enabled Structure-to-Sequence Transformer for Protein-Ligand Interaction Predictions. Nat. Mach. Intell..

[70] Jiang L., Jiang C., Yu X., Fu R., Jin S., Liu X. (2022). DeepTTA: A transformer-based model for predicting cancer drug response. Brief. Bioinform..

[71] Zhang O., Lin H., Zhang H., Zhao H., Huang Y., Hsieh C.-Y., Pan P., Hou T. (2024). Deep Lead Optimization: Leveraging Generative AI for Structural Modification. J. Am. Chem. Soc..

[72] Wang F., Feng X., Kong R., Chang S., Wang F., Feng X., Kong R., Chang S. (2023). Generating new protein sequences by using dense network and attention mechanism. Math. Biosci. Eng..

[73] Zeng X., Wang F., Luo Y., Kang S.-g., Tang J., Lightstone F.C., Fang E.F., Cornell W., Nussinov R., Cheng F. (2022). Deep generative molecular design reshapes drug discovery. Cell Rep. Med..

[74] Guimaraes G.L., Sanchez-Lengeling B., Outeiral C., Farias P.L.C., Aspuru-Guzik A. (2017). Objective-Reinforced Generative Adversarial Networks (ORGAN) for Sequence Generation Models. arXiv.

[75] Hu C., Li S., Yang C., Chen J., Xiong Y., Fan G., Liu H., Hong L. (2023). ScaffoldGVAE: Scaffold generation and hopping of drug molecules via a variational autoencoder based on multi-view graph neural networks. J. Cheminform..

[76] Wang M., Hsieh C.-Y., Wang J., Wang D., Weng G., Shen C., Yao X., Bing Z., Li H., Cao D. (2022). RELATION: A Deep Generative Model for Structure-Based De Novo Drug Design. J. Med. Chem..

[77] Tong X., Liu X., Tan X., Li X., Jiang J., Xiong Z., Xu T., Jiang H., Qiao N., Zheng M. (2021). Generative Models for De Novo Drug Design. J. Med. Chem..

[78] Ragoza M., Masuda T., Koes D.R. (2022). Generating 3D molecules conditional on receptor binding sites with deep generative models. Chem. Sci..

[79] Skalic M., Jiménez J., Sabbadin D., De Fabritiis G. (2019). Shape-Based Generative Modeling for de Novo Drug Design. J. Chem. Inf. Model..

[80] Guo D., Yang D., Zhang H., Song J.-M., Zhang R., Xu R., Zhu Q., Ma S., Wang P., DeepSeek-AI (2025). DeepSeek-R1: Incentivizing Reasoning Capability in LLMs via Reinforcement Learning. arXiv.

[81] Anthropic S. (2024). Model Card Addendum: Claude 3.5 Haiku and Upgraded Claude 3.5 Sonnet. https://assets.anthropic.com/m/1cd9d098ac3e6467/original/Claude-3-Model-Card-October-Addendum.pdf.

[82] Dubey A., Jauhri A., Pandey A., Kadian A., Al-Dahle A., Letman A., Mathur A., Schelten A., Yang A., Fan A. (2024). The Llama 3 Herd of Models. arXiv.

[83] El-Kishky A., Wei A., Saraiva A., Minaev B., Selsam D., Dohan D., Song F., Lightman H., Clavera I., Pachocki J.W. (2025). Competitive Programming with Large Reasoning Models. arXiv.

[84] Lin A., Ye J., Qi C., Zhu L., Mou W., Gan W., Zeng D., Tang B., Xiao M., Chu G. (2025). Bridging artificial intelligence and biological sciences: A comprehensive review of large language models in bioinformatics. Brief. Bioinform..

[85] Wu Z., Ramsundar B., Feinberg E.N., Gomes J., Geniesse C., Pappu A.S., Leswing K., Pande V. (2018). MoleculeNet: A benchmark for molecular machine learning. Chem. Sci..

[86] Huang K., Fu T., Gao W., Zhao Y., Roohani Y., Leskovec J., Coley C.W., Xiao C., Sun J., Zitnik M. (2022). Artificial intelligence foundation for therapeutic science. Nat. Chem. Biol..

[87] Xu L.-C., Tang M.-J., An J., Cao F., Qi Y. (2025). A unified pre-trained deep learning framework for cross-task reaction performance prediction and synthesis planning. Nat. Mach. Intell..

[88] He K., Zhang X., Ren S., Sun J. Deep Residual Learning for Image Recognition. Proceedings of the IEEE Conference on Computer Vision and Pattern Recognition (CVPR).

[89] Dou B., Zhu Z., Merkurjev E., Ke L., Chen L., Jiang J., Zhu Y., Liu J., Zhang B., Wei G.-W. (2023). Machine Learning Methods for Small Data Challenges in Molecular Science. Chem. Rev..

[90] Asfand-e-yar M., Hashir Q., Shah A.A., Malik H.A.M., Alourani A., Khalil W. (2024). Multimodal CNN-DDI: Using multimodal CNN for drug to drug interaction associated events. Sci. Rep..

[91] Chen S., Li T., Yang L., Zhai F., Jiang X., Xiang R., Ling G. (2022). Artificial intelligence-driven prediction of multiple drug interactions. Brief. Bioinform..

[92] Abbasi M., Carvalho F.G., Ribeiro B., Arrais J.P. (2024). Predicting drug activity against cancer through genomic profiles and SMILES. Artif. Intell. Med..

[93] Hochreiter S., Schmidhuber J. (1997). Long Short-Term Memory. Neural Comput..

[94] Isigkeit L., Hörmann T., Schallmayer E., Scholz K., Lillich F.F., Ehrler J.H.M., Hufnagel B., Büchner J., Marschner J.A., Pabel J. (2024). Automated design of multi-target ligands by generative deep learning. Nat. Commun..

[95] Huang K., Chandak P., Wang Q., Havaldar S., Vaid A., Leskovec J., Nadkarni G.N., Glicksberg B.S., Gehlenborg N., Zitnik M. (2024). A foundation model for clinician-centered drug repurposing. Nat. Med..

[96] Lim J., Ryu S., Park K., Choe Y.J., Ham J., Kim W.Y. (2019). Predicting Drug–Target Interaction Using a Novel Graph Neural Network with 3D Structure-Embedded Graph Representation. J. Chem. Inf. Model..

[97] Sun Y., Li Y.Y., Leung C.K., Hu P. (2024). iNGNN-DTI: Prediction of drug–target interaction with interpretable nested graph neural network and pretrained molecule models. Bioinformatics.

[98] Zhang Z., Chen L., Zhong F., Wang D., Jiang J., Zhang S., Jiang H., Zheng M., Li X. (2022). Graph neural network approaches for drug-target interactions. Curr. Opin. Struct. Biol..

[99] Ng S.S.S., Lu Y. (2023). Evaluating the Use of Graph Neural Networks and Transfer Learning for Oral Bioavailability Prediction. J. Chem. Inf. Model..

[100] Yang Z., Zhong W., Zhao L., Chen C.Y.-C. (2022). MGraphDTA: Deep multiscale graph neural network for explainable drug–target binding affinity prediction. Chem. Sci..

[101] Li P., Hua L., Ma Z., Hu W., Liu Y., Zhu J. (2024). Conformalized Graph Learning for Molecular ADMET Property Prediction and Reliable Uncertainty Quantification. J. Chem. Inf. Model..

[102] Heid E., Greenman K.P., Chung Y., Li S.-C., Graff D.E., Vermeire F.H., Wu H., Green W.H., McGill C.J. (2024). Chemprop: A Machine Learning Package for Chemical Property Prediction. J. Chem. Inf. Model..

[103] Bai P., Miljković F., John B., Lu H. (2023). Interpretable bilinear attention network with domain adaptation improves drug–target prediction. Nat. Mach. Intell..

[104] Vaswani A. (2017). Attention is all you need. Adv. Neural Inf. Process. Syst..

[105] Radford A., Narasimhan K. (2018). Improving Language Understanding by Generative Pre-Training. https://www.semanticscholar.org/paper/Improving-Language-Understanding-by-Generative-Radford-Narasimhan/cd18800a0fe0b668a1cc19f2ec95b5003d0a5035.

[106] Devlin J., Chang M.-W., Lee K., Toutanova K. BERT: Pre-training of Deep Bidirectional Transformers for Language Understanding. Proceedings of the 2019 Conference of the North American Chapter of the Association for Computational Linguistics: Human Language Technologies, Volume 1 (Long and Short Papers).

[107] Mao J., Wang J., Zeb A., Cho K.-H., Jin H., Kim J., Lee O., Wang Y., No K.T. (2024). Transformer-Based Molecular Generative Model for Antiviral Drug Design. J. Chem. Inf. Model..

[108] Achiam J., Adler S., Agarwal S., Ahmad L., Akkaya I., Aleman F.L., Almeida D., Altenschmidt J., Altman S., OpenAi (2024). GPT-4 Technical Report. arXiv.

[109] Brown T.B., Mann B., Ryder N., Subbiah M., Kaplan J., Dhariwal P., Neelakantan A., Shyam P., Sastry G., Askell A. Language models are few-shot learners. Proceedings of the 34th International Conference on Neural Information Processing Systems.

[110] Liu S., Wang J., Yang Y., Wang C., Liu L., Guo H., Xiao C. (2023). Chatgpt-powered conversational drug editing using retrieval and domain feedback. arXiv.

[111] Greenblatt J.F., Alberts B.M., Krogan N.J. (2024). Discovery and significance of protein-protein interactions in health and disease. Cell.

[112] Lim Y., Tamayo-Orrego L., Schmid E., Tarnauskaite Z., Kochenova O.V., Gruar R., Muramatsu S., Lynch L., Schlie A.V., Carroll P.L. (2023). In silico protein interaction screening uncovers DONSON’s role in replication initiation. Science.

[113] Lin Z., Akin H., Rao R., Hie B., Zhu Z., Lu W., Smetanin N., Verkuil R., Kabeli O., Shmueli Y. (2023). Evolutionary-scale prediction of atomic-level protein structure with a language model. Science.

[114] Senior A.W., Evans R., Jumper J., Kirkpatrick J., Sifre L., Green T., Qin C., Žídek A., Nelson A.W.R., Bridgland A. (2020). Improved protein structure prediction using potentials from deep learning. Nature.

[115] Jumper J., Evans R., Pritzel A., Green T., Figurnov M., Ronneberger O., Tunyasuvunakool K., Bates R., Žídek A., Potapenko A. (2021). Highly accurate protein structure prediction with AlphaFold. Nature.

[116] Evans R., O’Neill M., Pritzel A., Antropova N., Senior A., Green T., Žídek A., Bates R., Blackwell S., Yim J. (2021). Protein complex prediction with AlphaFold-Multimer. bioRxiv.

[117] Abramson J., Adler J., Dunger J., Evans R., Green T., Pritzel A., Ronneberger O., Willmore L., Ballard A.J., Bambrick J. (2024). Accurate structure prediction of biomolecular interactions with AlphaFold 3. Nature.

[118] Díaz-Holguín A., Saarinen M., Vo D.D., Sturchio A., Branzell N., Cabeza de Vaca I., Hu H., Mitjavila-Domènech N., Lindqvist A., Baranczewski P. (2024). AlphaFold accelerated discovery of psychotropic agonists targeting the trace amine–associated receptor 1. Sci. Adv..

[119] Bernatavicius A., Šícho M., Janssen A.P.A., Hassen A.K., Preuss M., van Westen G.J.P. (2024). AlphaFold Meets De Novo Drug Design: Leveraging Structural Protein Information in Multitarget Molecular Generative Models. J. Chem. Inf. Model..

[120] Ren F., Ding X., Zheng M., Korzinkin M., Cai X., Zhu W., Mantsyzov A., Aliper A., Aladinskiy V., Cao Z. (2023). AlphaFold accelerates artificial intelligence powered drug discovery: Efficient discovery of a novel CDK20 small molecule inhibitor. Chem. Sci..

[121] Michaud J.M., Madani A., Fraser J.S. (2022). A language model beats alphafold2 on orphans. Nat. Biotechnol..

[122] Baek M., DiMaio F., Anishchenko I., Dauparas J., Ovchinnikov S., Lee G.R., Wang J., Cong Q., Kinch L.N., Schaeffer R.D. (2021). Accurate prediction of protein structures and interactions using a three-track neural network. Science.

[123] Du Z., Su H., Wang W., Ye L., Wei H., Peng Z., Anishchenko I., Baker D., Yang J. (2021). The trRosetta server for fast and accurate protein structure prediction. Nat. Protoc..

[124] Zhou Y., Zhang Y., Lian X., Li F., Wang C., Zhu F., Qiu Y., Chen Y. (2022). Therapeutic target database update 2022: Facilitating drug discovery with enriched comparative data of targeted agents. Nucleic Acids Res..

[125] Finan C., Gaulton A., Kruger F.A., Lumbers R.T., Shah T., Engmann J., Galver L., Kelley R., Karlsson A., Santos R. (2017). The druggable genome and support for target identification and validation in drug development. Sci. Transl. Med..

[126] Kana O., Brylinski M. (2019). Elucidating the druggability of the human proteome with eFindSite. J. Comput. Aided Mol. Des..

[127] Li X., Wang S., Xie Y., Jiang H., Guo J., Wang Y., Peng Z., Hu M., Wang M., Wang J. (2023). Deacetylation induced nuclear condensation of HP1gamma promotes multiple myeloma drug resistance. Nat. Commun..

[128] Wang Y., Gao S., Chen L., Liu S., Ma J., Cao Z., Li Q. (2022). DUT enhances drug resistance to proteasome inhibitors via promoting mitochondrial function in multiple myeloma. Carcinogenesis.

[129] Qi T.F., Tang F., Yin J., Miao W., Wang Y. (2023). Parallel-reaction monitoring revealed altered expression of a number of epitranscriptomic reader, writer, and eraser proteins accompanied with colorectal cancer metastasis. Proteomics.

[130] Nidhi S., Anand U., Oleksak P., Tripathi P., Lal J.A., Thomas G., Kuca K., Tripathi V. (2021). Novel CRISPR-Cas Systems: An Updated Review of the Current Achievements, Applications, and Future Research Perspectives. Int. J. Mol. Sci..

[131] Ramkumar P., Abarientos A.B., Tian R., Seyler M., Leong J.T., Chen M., Choudhry P., Hechler T., Shah N., Wong S.W. (2020). CRISPR-based screens uncover determinants of immunotherapy response in multiple myeloma. Blood Adv..

[132] Raivola J., Dini A., Karvonen H., Piki E., Salokas K., Niininen W., Kaleva L., Zhang K., Arjama M., Gudoityte G. (2022). Multiomics characterization implicates PTK7 in ovarian cancer EMT and cell plasticity and offers strategies for therapeutic intervention. Cell Death Dis..

[133] Gulfidan G., Soylu M., Demirel D., Erdonmez H.B.C., Beklen H., Ozbek Sarica P., Arga K.Y., Turanli B. (2022). Systems biomarkers for papillary thyroid cancer prognosis and treatment through multi-omics networks. Arch Biochem. Biophys..

[134] Assum I., Krause J., Scheinhardt M.O., Muller C., Hammer E., Borschel C.S., Volker U., Conradi L., Geelhoed B., Zeller T. (2022). Tissue-specific multi-omics analysis of atrial fibrillation. Nat. Commun..

[135] Abell N.S., DeGorter M.K., Gloudemans M.J., Greenwald E., Smith K.S., He Z., Montgomery S.B. (2022). Multiple causal variants underlie genetic associations in humans. Science.

[136] Namba S., Konuma T., Wu K.H., Zhou W., Global Biobank Meta-analysis I., Okada Y. (2022). A practical guideline of genomics-driven drug discovery in the era of global biobank meta-analysis. Cell Genom..

[137] Deelen J., Evans D.S., Arking D.E., Tesi N., Nygaard M., Liu X., Wojczynski M.K., Biggs M.L., van der Spek A., Atzmon G. (2021). Publisher Correction: A meta-analysis of genome-wide association studies identifies multiple longevity genes. Nat. Commun..

[138] Vamathevan J., Clark D., Czodrowski P., Dunham I., Ferran E., Lee G., Li B., Madabhushi A., Shah P., Spitzer M. (2019). Applications of machine learning in drug discovery and development. Nat. Rev. Drug. Discov..

[139] Lo Y.C., Senese S., Damoiseaux R., Torres J.Z. (2016). 3D Chemical Similarity Networks for Structure-Based Target Prediction and Scaffold Hopping. ACS Chem. Biol..

[140] Wolber G., Seidel T., Bendix F., Langer T. (2008). Molecule-pharmacophore superpositioning and pattern matching in computational drug design. Drug Discov. Today.

[141] Mamoshina P., Volosnikova M., Ozerov I.V., Putin E., Skibina E., Cortese F., Zhavoronkov A. (2018). Machine Learning on Human Muscle Transcriptomic Data for Biomarker Discovery and Tissue-Specific Drug Target Identification. Front. Genet..

[142] Theodoris C.V., Xiao L., Chopra A., Chaffin M.D., Al Sayed Z.R., Hill M.C., Mantineo H., Brydon E.M., Zeng Z., Liu X.S. (2023). Transfer learning enables predictions in network biology. Nature.

[143] Kang B., Fan R., Cui C., Cui Q. (2025). Comprehensive prediction and analysis of human protein essentiality based on a pretrained large language model. Nat. Comput. Sci..

[144] Zhang S., Cooper-Knock J., Weimer A.K., Shi M., Moll T., Marshall J.N.G., Harvey C., Nezhad H.G., Franklin J., Souza C.D.S. (2022). Genome-wide identification of the genetic basis of amyotrophic lateral sclerosis. Neuron.

[145] Zeng X., Zhu S., Lu W., Liu Z., Huang J., Zhou Y., Fang J., Huang Y., Guo H., Li L. (2020). Target identification among known drugs by deep learning from heterogeneous networks. Chem. Sci..

[146] Zhou L., Lian G., Zhou T., Cai Z., Yang S., Li W., Cheng L., Ye Y., He M., Lu J. (2025). Palmitoylation of GPX4 via the targetable ZDHHC8 determines ferroptosis sensitivity and antitumor immunity. Nat. Cancer.

[147] Yang Y., Yao K., Repasky M.P., Leswing K., Abel R., Shoichet B.K., Jerome S.V. (2021). Efficient Exploration of Chemical Space with Docking and Deep Learning. J. Chem. Theory Comput..

[148] Singh R., Sledzieski S., Bryson B., Cowen L., Berger B. (2023). Contrastive learning in protein language space predicts interactions between drugs and protein targets. Proc. Natl. Acad. Sci. USA.

[149] Schade A.E., Perurena N., Yang Y., Rodriguez C.L., Krishnan A., Gardner A., Loi P., Xu Y., Nguyen V.T.M., Mastellone G.M. (2024). AKT and EZH2 inhibitors kill TNBCs by hijacking mechanisms of involution. Nature.

[150] Gentile F., Yaacoub J.C., Gleave J., Fernandez M., Ton A.T., Ban F., Stern A., Cherkasov A. (2022). Artificial intelligence-enabled virtual screening of ultra-large chemical libraries with deep docking. Nat. Protoc..

[151] Acharya A., Agarwal R., Baker M.B., Baudry J., Bhowmik D., Boehm S., Byler K.G., Chen S.Y., Coates L., Cooper C.J. (2020). Supercomputer-Based Ensemble Docking Drug Discovery Pipeline with Application to Covid-19. J. Chem. Inf. Model..

[152] Puszkarska A.M., Taddese B., Revell J., Davies G., Field J., Hornigold D.C., Buchanan A., Vaughan T.J., Colwell L.J. (2024). Machine learning designs new GCGR/GLP-1R dual agonists with enhanced biological potency. Nat. Chem..

[153] Trinh T.C., Falson P., Tran-Nguyen V.K., Boumendjel A. (2025). Ligand-Based Drug Discovery Leveraging State-of-the-Art Machine Learning Methodologies Exemplified by Cdr1 Inhibitor Prediction. J. Chem. Inf. Model..

[154] Hansson F.G., Madsen N.G., Hansen L.G., Jakociunas T., Lengger B., Keasling J.D., Jensen M.K., Acevedo-Rocha C.G., Jensen E.D. (2025). Labels as a feature: Network homophily for systematically annotating human GPCR drug-target interactions. Nat. Commun..

[155] Hadipour H., Li Y.Y., Sun Y., Deng C., Lac L., Davis R., Cardona S.T., Hu P. (2025). GraphBAN: An inductive graph-based approach for enhanced prediction of compound-protein interactions. Nat. Commun..

[156] Lu Z., Song G., Zhu H., Lei C., Sun X., Wang K., Qin L., Chen Y., Tang J., Li M. (2025). DTIAM: A unified framework for predicting drug-target interactions, binding affinities and drug mechanisms. Nat. Commun..

[157] Liu X., Li Q., Yan X., Wang L., Qiu J., Yao X., Liu H. (2025). A Specialized and Enhanced Deep Generation Model for Active Molecular Design Targeting Kinases Guided by Affinity Prediction Models and Reinforcement Learning. J. Chem. Inf. Model..

[158] Yang H., Chen Y., Zuo Y., Deng Z., Pan X., Shen H.B., Choi K.S., Yu D.J. (2024). MINDG: A drug-target interaction prediction method based on an integrated learning algorithm. Bioinformatics.

[159] Moon S., Hwang S.-Y., Lim J., Kim W.Y. (2024). PIGNet2: A versatile deep learning-based protein–ligand interaction prediction model for binding affinity scoring and virtual screening. Digit. Discov..

[160] Cai H., Shen C., Jian T., Zhang X., Chen T., Han X., Yang Z., Dang W., Hsieh C.Y., Kang Y. (2024). CarsiDock: A deep learning paradigm for accurate protein-ligand docking and screening based on large-scale pre-training. Chem. Sci..

[161] Jin W., Stokes J.M., Eastman R.T., Itkin Z., Zakharov A.V., Collins J.J., Jaakkola T.S., Barzilay R. (2021). Deep learning identifies synergistic drug combinations for treating COVID-19. Proc. Natl. Acad. Sci. USA.

[162] Liu G., Catacutan D.B., Rathod K., Swanson K., Jin W., Mohammed J.C., Chiappino-Pepe A., Syed S.A., Fragis M., Rachwalski K. (2023). Deep learning-guided discovery of an antibiotic targeting *Acinetobacter baumannii*. Nat. Chem. Biol..

[163] Guo X., Zhao X., Lu X., Zhao L., Zeng Q., Chen F., Zhang Z., Xu M., Feng S., Fan T. (2024). A deep learning-driven discovery of berberine derivatives as novel antibacterial against multidrug-resistant *Helicobacter pylori*. Signal Transduct. Target. Ther..

[164] Liu X.-W., Shi T.-Y., Gao D., Ma C.-Y., Lin H., Yan D., Deng K.-J. (2023). iPADD: A Computational Tool for Predicting Potential Antidiabetic Drugs Using Machine Learning Algorithms. J. Chem. Inf. Model..

[165] Gerdes H., Casado P., Dokal A., Hijazi M., Akhtar N., Osuntola R., Rajeeve V., Fitzgibbon J., Travers J., Britton D. (2021). Drug ranking using machine learning systematically predicts the efficacy of anti-cancer drugs. Nat. Commun..

[166] Feng B., Liu Z., Huang N., Xiao Z., Zhang H., Mirzoyan S., Xu H., Hao J., Xu Y., Zhang M. (2024). A bioactivity foundation model using pairwise meta-learning. Nat. Mach. Intell..

[167] Duan Y., Yang X., Zeng X., Wang W., Deng Y., Cao D. (2024). Enhancing Molecular Property Prediction through Task-Oriented Transfer Learning: Integrating Universal Structural Insights and Domain-Specific Knowledge. J. Med. Chem..

[168] Cai H., Zhang H., Zhao D., Wu J., Wang L. (2022). FP-GNN: A versatile deep learning architecture for enhanced molecular property prediction. Brief. Bioinform..

[169] Ahmad W., Simon E., Chithrananda S., Grand G., Ramsundar B. (2022). Chemberta-2: Towards chemical foundation models. arXiv.

[170] Zhou G., Gao Z., Ding Q., Zheng H., Xu H., Wei Z., Zhang L., Ke G. (2023). Uni-mol: A universal 3d molecular representation learning framework. ChemRxiv.

[171] Chang J., Ye J.C. (2024). Bidirectional generation of structure and properties through a single molecular foundation model. Nat. Commun..

[172] Ross J., Belgodere B., Chenthamarakshan V., Padhi I., Mroueh Y., Das P. (2022). Large-scale chemical language representations capture molecular structure and properties. Nat. Mach. Intell..

[173] Virshup A.M., Contreras-García J., Wipf P., Yang W., Beratan D.N. (2013). Stochastic Voyages into Uncharted Chemical Space Produce a Representative Library of All Possible Drug-Like Compounds. J. Am. Chem. Soc..

[174] Polishchuk P.G., Madzhidov T.I., Varnek A. (2013). Estimation of the size of drug-like chemical space based on GDB-17 data. J. Comput.-Aided Mol. Des..

[175] Tropsha A., Isayev O., Varnek A., Schneider G., Cherkasov A. (2024). Integrating QSAR modelling and deep learning in drug discovery: The emergence of deep QSAR. Nat. Rev. Drug Discov..

[176] Carvalho Martins L., Cino E.A., Ferreira R.S. (2021). PyAutoFEP: An Automated Free Energy Perturbation Workflow for GROMACS Integrating Enhanced Sampling Methods. J. Chem. Theory Comput..

[177] Schneuing A., Harris C., Du Y., Didi K., Jamasb A., Igashov I., Du W., Gomes C., Blundell T.L., Lio P. (2024). Structure-based drug design with equivariant diffusion models. Nat. Comput. Sci..

[178] Li P., Zhang K., Liu T., Lu R., Chen Y., Yao X., Gao L., Zeng X. (2024). A deep learning approach for rational ligand generation with toxicity control via reactive building blocks. Nat. Comput. Sci..

[179] Hu Q., Sun C., He H., Xu J., Liu D., Zhang W., Shi S., Zhang K., Li H. (2025). Target-aware 3D molecular generation based on guided equivariant diffusion. Nat. Commun..

[180] Godinez W.J., Ma E.J., Chao A.T., Pei L., Skewes-Cox P., Canham S.M., Jenkins J.L., Young J.M., Martin E.J., Guiguemde W.A. (2022). Design of potent antimalarials with generative chemistry. Nat. Mach. Intell..

[181] Swanson K., Liu G., Catacutan D.B., Arnold A., Zou J., Stokes J.M. (2024). Generative AI for designing and validating easily synthesizable and structurally novel antibiotics. Nat. Mach. Intell..

[182] Wu K., Xia Y., Deng P., Liu R., Zhang Y., Guo H., Cui Y., Pei Q., Wu L., Xie S. (2024). TamGen: Drug design with target-aware molecule generation through a chemical language model. Nat. Commun..

[183] Skinnider M.A., Stacey R.G., Wishart D.S., Foster L.J. (2021). Chemical language models enable navigation in sparsely populated chemical space. Nat. Mach. Intell..

[184] Flam-Shepherd D., Zhu K., Aspuru-Guzik A. (2022). Language models can learn complex molecular distributions. Nat. Commun..

[185] Grisoni F. (2023). Chemical language models for de novo drug design: Challenges and opportunities. Curr. Opin. Struct. Biol..

[186] Tong X., Qu N., Kong X., Ni S., Zhou J., Wang K., Zhang L., Wen Y., Shi J., Zhang S. (2024). Deep representation learning of chemical-induced transcriptional profile for phenotype-based drug discovery. Nat. Commun..

[187] Tan Y., Dai L., Huang W., Guo Y., Zheng S., Lei J., Chen H., Yang Y. (2022). DRlinker: Deep Reinforcement Learning for Optimization in Fragment Linking Design. J. Chem. Inf. Model..

[188] Guo J., Knuth F., Margreitter C., Janet J.P., Papadopoulos K., Engkvist O., Patronov A. (2023). Link-INVENT: Generative linker design with reinforcement learning. Digit. Discov..

[189] Zheng S., Lei Z., Ai H., Chen H., Deng D., Yang Y. (2021). Deep scaffold hopping with multimodal transformer neural networks. J. Cheminform..

[190] Bagal V., Aggarwal R., Vinod P.K., Priyakumar U.D. (2022). MolGPT: Molecular Generation Using a Transformer-Decoder Model. J. Chem. Inf. Model..

[191] Arus-Pous J., Patronov A., Bjerrum E.J., Tyrchan C., Reymond J.L., Chen H., Engkvist O. (2020). SMILES-based deep generative scaffold decorator for de-novo drug design. J. Cheminform..

[192] Langevin M., Minoux H., Levesque M., Bianciotto M. (2020). Scaffold-Constrained Molecular Generation. J. Chem. Inf. Model..

[193] Fialkova V., Zhao J., Papadopoulos K., Engkvist O., Bjerrum E.J., Kogej T., Patronov A. (2022). LibINVENT: Reaction-based Generative Scaffold Decoration for in Silico Library Design. J. Chem. Inf. Model..

[194] Xie J., Chen S., Lei J., Yang Y. (2024). DiffDec: Structure-Aware Scaffold Decoration with an End-to-End Diffusion Model. J. Chem. Inf. Model..

[195] Green H., Koes D.R., Durrant J.D. (2021). DeepFrag: A deep convolutional neural network for fragment-based lead optimization. Chem. Sci..

[196] Hadfield T.E., Imrie F., Merritt A., Birchall K., Deane C.M. (2022). Incorporating Target-Specific Pharmacophoric Information into Deep Generative Models for Fragment Elaboration. J. Chem. Inf. Model..

[197] Imrie F., Hadfield T.E., Bradley A.R., Deane C.M. (2021). Deep generative design with 3D pharmacophoric constraints. Chem. Sci..

[198] Liu X., Ye K., van Vlijmen H.W.T., AP I.J., van Westen G.J.P. (2023). DrugEx v3: Scaffold-constrained drug design with graph transformer-based reinforcement learning. J. Cheminform..

[199] Loeffler H.H., He J., Tibo A., Janet J.P., Voronov A., Mervin L.H., Engkvist O. (2024). Reinvent 4: Modern AI-driven generative molecule design. J. Cheminform..

[200] Wang M., Li S., Wang J., Zhang O., Du H., Jiang D., Wu Z., Deng Y., Kang Y., Pan P. (2024). ClickGen: Directed exploration of synthesizable chemical space via modular reactions and reinforcement learning. Nat. Commun..

[201] Segler M.H.S., Preuss M., Waller M.P. (2018). Planning chemical syntheses with deep neural networks and symbolic AI. Nature.

[202] Thakkar A., Chadimová V., Bjerrum E.J., Engkvist O., Reymond J.-L. (2021). Retrosynthetic accessibility score (RAscore)—rapid machine learned synthesizability classification from AI driven retrosynthetic planning. Chem. Sci..

[203] Nippa D.F., Atz K., Hohler R., Muller A.T., Marx A., Bartelmus C., Wuitschik G., Marzuoli I., Jost V., Wolfard J. (2024). Enabling late-stage drug diversification by high-throughput experimentation with geometric deep learning. Nat. Chem..

[204] Lu J., Zhang Y. (2022). Unified Deep Learning Model for Multitask Reaction Predictions with Explanation. J. Chem. Inf. Model..

[205] Li B., Su S., Zhu C., Lin J., Hu X., Su L., Yu Z., Liao K., Chen H. (2023). A deep learning framework for accurate reaction prediction and its application on high-throughput experimentation data. J. Cheminform.

[206] Cong S., Zhang M., Song Y., Chang S., Tian J., Zeng H., Ji H. (2025). Graph-sequence enhanced transformer for template-free prediction of natural product biosynthesis. Patterns.

[207] Lee A.A., Yang Q., Sresht V., Bolgar P., Hou X., Klug-McLeod J.L., Butler C.R. (2019). Molecular Transformer unifies reaction prediction and retrosynthesis across pharma chemical space. Chem. Commun..

[208] Zhao P.C., Wei X.X., Wang Q., Wang Q.H., Li J.N., Shang J., Lu C., Shi J.Y. (2025). Single-step retrosynthesis prediction via multitask graph representation learning. Nat. Commun..

[209] Chen S., Jung Y. (2021). Deep Retrosynthetic Reaction Prediction using Local Reactivity and Global Attention. JACS Au.

[210] Ucak U.V., Ashyrmamatov I., Ko J., Lee J. (2022). Retrosynthetic reaction pathway prediction through neural machine translation of atomic environments. Nat. Commun..

[211] Fang L., Li J., Zhao M., Tan L., Lou J.G. (2023). Single-step retrosynthesis prediction by leveraging commonly preserved substructures. Nat. Commun..

[212] Lin Z., Yin S., Shi L., Zhou W., Zhang Y.J. (2023). G2GT: Retrosynthesis Prediction with Graph-to-Graph Attention Neural Network and Self-Training. J. Chem. Inf. Model..

[213] Mao K., Xiao X., Xu T., Rong Y., Huang J., Zhao P. (2021). Molecular graph enhanced transformer for retrosynthesis prediction. Neurocomputing.

[214] Tu Z., Coley C.W. (2022). Permutation Invariant Graph-to-Sequence Model for Template-Free Retrosynthesis and Reaction Prediction. J. Chem. Inf. Model..

[215] Wang X., Li Y., Qiu J., Chen G., Liu H., Liao B., Hsieh C.-Y., Yao X. (2021). Retroprime: A diverse, plausible and transformer-based method for single-step retrosynthesis predictions. Chem. Eng. J..

[216] Beckers M., Sturm N., Sirockin F., Fechner N., Stiefl N. (2023). Prediction of Small-Molecule Developability Using Large-Scale In Silico ADMET Models. J. Med. Chem..

[217] Vangala S.R., Krishnan S.R., Bung N., Srinivasan R., Roy A. (2023). pBRICS: A Novel Fragmentation Method for Explainable Property Prediction of Drug-like Small Molecules. J. Chem. Inf. Model..

[218] Jamrozik E., Śmieja M., Podlewska S. (2024). ADMET-PrInt: Evaluation of ADMET Properties: Prediction and Interpretation. J. Chem. Inf. Model..

[219] Mamada H., Takahashi M., Ogino M., Nomura Y., Uesawa Y. (2023). Predictive Models Based on Molecular Images and Molecular Descriptors for Drug Screening. ACS Omega.

[220] Komissarov L., Manevski N., Groebke Zbinden K., Schindler T., Zitnik M., Sach-Peltason L. (2024). Actionable Predictions of Human Pharmacokinetics at the Drug Design Stage. Mol. Pharm..

[221] Niu Z., Xiao X., Wu W., Cai Q., Jiang Y., Jin W., Wang M., Yang G., Kong L., Jin X. (2024). PharmaBench: Enhancing ADMET benchmarks with large language models. Sci. Data.

[222] Swanson K., Walther P., Leitz J., Mukherjee S., Wu J.C., Shivnaraine R.V., Zou J. (2024). ADMET-AI: A machine learning ADMET platform for evaluation of large-scale chemical libraries. Bioinformatics.

[223] Daina A., Michielin O., Zoete V. (2017). SwissADME: A free web tool to evaluate pharmacokinetics, drug-likeness and medicinal chemistry friendliness of small molecules. Sci. Rep..

[224] Fu L., Shi S., Yi J., Wang N., He Y., Wu Z., Peng J., Deng Y., Wang W., Wu C. (2024). ADMETlab 3.0: An updated comprehensive online ADMET prediction platform enhanced with broader coverage, improved performance, API functionality and decision support. Nucleic Acids Res..

[225] Pires D.E., Blundell T.L., Ascher D.B. (2015). pkCSM: Predicting Small-Molecule Pharmacokinetic and Toxicity Properties Using Graph-Based Signatures. J. Med. Chem..

[226] Schyman P., Liu R., Desai V., Wallqvist A. (2017). vNN Web Server for ADMET Predictions. Front. Pharmacol..

[227] Yi J.C., Yang Z.Y., Zhao W.T., Yang Z.J., Zhang X.C., Wu C.K., Lu A.P., Cao D.S. (2024). ChemMORT: An automatic ADMET optimization platform using deep learning and multi-objective particle swarm optimization. Brief. Bioinform..

[228] Zhang K., Yang X., Wang Y., Yu Y., Huang N., Li G., Li X., Wu J.C., Yang S. (2025). Artificial intelligence in drug development. Nat. Med..

[229] Ji Z., Lee N., Frieske R., Yu T., Su D., Xu Y., Ishii E., Bang Y.J., Madotto A., Fung P. (2023). Survey of Hallucination in Natural Language Generation. ACM Comput. Surv..

[230] Wellawatte G.P., Gandhi H.A., Seshadri A., White A.D. (2023). A Perspective on Explanations of Molecular Prediction Models. J. Chem. Theory Comput..

[231] Frye L., Bhat S., Akinsanya K., Abel R. (2021). From computer-aided drug discovery to computer-driven drug discovery. Drug Discov. Today Technol..

